# A novel Ca2+-binding protein that can rapidly transduce auxin responses during root growth

**DOI:** 10.1371/journal.pbio.3000085

**Published:** 2019-07-11

**Authors:** Ora Hazak, Elad Mamon, Meirav Lavy, Hasana Sternberg, Smrutisanjita Behera, Ina Schmitz-Thom, Daria Bloch, Olga Dementiev, Itay Gutman, Tomer Danziger, Netanel Schwarz, Anas Abuzeineh, Keithanne Mockaitis, Mark Estelle, Joel A. Hirsch, Jörg Kudla, Shaul Yalovsky

**Affiliations:** 1 School of Plant Sciences and Food Security, Tel Aviv University, Tel Aviv, Israel; 2 Institute of Biology and Biotechnology of Plants, University of Münster, Münster, Germany; 3 Department of Biology, University of Indiana, Bloomington, Indiana, United States of America; 4 Howard Hughes Medical Institute and Division of Biology, University of California, San Diego, La Jolla, California, United States of America; 5 Department of Biochemistry and Molecular Biology, Tel Aviv University, Tel Aviv, Israel; University of California, Riverside, UNITED STATES

## Abstract

Signaling cross talks between auxin, a regulator of plant development, and Ca^2+^, a universal second messenger, have been proposed to modulate developmental plasticity in plants. However, the underlying molecular mechanisms are largely unknown. Here, we report that in *Arabidopsis* roots, auxin elicits specific Ca^2+^ signaling patterns that spatially coincide with the expression pattern of auxin-regulated genes. We have identified the single EF-hand Ca^2+^-binding protein Ca^2+^-dependent modulator of ICR1 (CMI1) as an interactor of the Rho of plants (ROP) effector interactor of constitutively active ROP (ICR1). CMI1 expression is directly up-regulated by auxin, whereas the loss of function of CMI1 associates with the repression of auxin-induced Ca^2+^ increases in the lateral root cap and vasculature, indicating that CMI1 represses early auxin responses. In agreement, *cmi1* mutants display an increased auxin response including shorter primary roots, longer root hairs, longer hypocotyls, and altered lateral root formation. Binding to ICR1 affects subcellular localization of CMI1 and its function. The interaction between CMI1 and ICR1 is Ca^2+^-dependent and involves a conserved hydrophobic pocket in CMI1 and calmodulin binding-like domain in ICR1. Remarkably, CMI1 is monomeric in solution and in vitro changes its secondary structure at cellular resting Ca^2+^ concentrations ranging between 10^−9^ and 10^−8^ M. Hence, CMI1 is a Ca^2+^-dependent transducer of auxin-regulated gene expression, which can function in a cell-specific fashion at steady-state as well as at elevated cellular Ca^2+^ levels to regulate auxin responses.

## Abstract 2

An auxin-regulated calcium-dependent signal transducer, CMI1, regulates auxin responses and root development, providing a basis for cross talk between auxin and calcium signaling in plants.

## Introduction

The plant hormone auxin regulates diverse developmental and physiological processes, serving as a morphogen that creates local maxima and gradients [1]. Within plant roots, auxin accumulates at the growing tip, with a maximum at the quiescent center (QC) and the below organized columella cells. It is well documented that this accumulation happens early during embryo development and is essential for root meristem maintenance and root growth [1–5]. Interestingly, this long-term auxin accumulation is translated to gradients of transcription factors PLETHORA (PLT) genes and specific expression pattern of auxin response factors (ARFs) and their Aux/indole-3-acetic acid (IAA) inhibitors that have been shown to maintain the activity of root stem cells [5–8]. In addition, nontranscriptional responses to auxin have been suspected, but it remains mysterious how they are regulated by auxin gradients at the root tip.

Auxin facilitates its own accumulation in the plant tissues by modulating the polar auxin transport and the local biosynthesis rates [9–11]. Auxin transport depends on AUX1/Like AUX 1 (LAX) auxin influx transporters [12], PINFORMED (PIN) proteins, ATP-binding cassette B (ABCB) auxin efflux transporters [13,14], and at low-nitrogen conditions by nitrate transporter 1.1 (NRT1.1) NO_3_^−^ influx transporter [15,16]. The AGCVIII kinase PINOID (PID)—which regulates PIN1, PIN2, and PIN3 distribution [17,18] and PIN-mediated auxin transport [19]—interacts with two EF-hand Ca^2+^ binding proteins, TOUCH3 (TCH3), and PID-binding protein 1 (PBP1) [20]. Moreover, PID overexpression–induced root meristem collapse was reduced by treatments with LaCl_3_, a Ca^2+^ channel inhibitor suggesting the requirement of Ca^2+^ for PID function and, consequently, PIN regulation [20]. However, it is not known yet how the Ca^2+^-binding proteins TCH3 and PBP1 affect PID function.

From the molecular perspective, auxin operates as a "molecular glue" mediating the binding of the Aux/IAA transcriptional repressors to the Skp Cullin F-box transport inhibitor response 1 (SCF^TIR1/AFB^) E3 ubiquitin ligase complex, resulting in polyubiquitilation and proteasomal degradation of the Aux/IAAs, leading to activation of the ARF transcriptional regulators [1,21–23]. In addition, auxin induces rapid transcription-independent responses such as membrane depolarization and Ca^2+^ influx by mechanisms that depend on auxin perception by TIR1/AFB [24–28]. Signaling cross talks between auxin and Ca^2+^ have been proposed to modulate developmental plasticity in plants [24,29–31].

In plants, diverse environmental stimuli induce elevations in cytosolic Ca^2+^ from steady-state levels of around 10^−7^ M to 10^−6^ to 10^−5^ M, which are in turn transduced by plethora of Ca^2+^-binding proteins. The majority of these proteins—including calmodulins (CaMs), CaM-like (CML), Ca^2+^-dependent kinases (CDPKs), and calcineurin B-like (CBL)—contain two or more Ca^2+^-binding EF-hand motifs. CaMs, CMLs, CDPKs, and CBLs bind Ca^2+^ only when its concentrations are at the μM range and hence function as Ca^2+^ sensors that transduce response only when Ca^2+^ levels increase from steady-state levels [32,33]. EF-hand proteins that can bind Ca^2+^ at concentrations lower than 10^−7^ M have not been extensively studied. Although such proteins do not likely function as Ca^2+^ sensors, they can function as response transducers directly dependent on their expression levels.

Ca^2+^ has been named “the missing link in auxin action” [34]. AUX1-dependent auxin influx in root and root hairs induces cyclic nucleotide gated channel 14 (CNGC14)- and TIR1/AFB-dependent Ca^2+^ signaling within seconds that in turn affects downstream auxin signaling [24–26]. CNGC14 function is required in response to gravity stimulus [25], indicating that function involves Ca^2+^ signaling. Given the rapid Ca^2+^-associated response that is induced by auxin, the existence of auxin up-regulated Ca^2+^-binding proteins that bind Ca^2+^ at its steady-state levels and can therefore transduce Ca^2+^ responses upon their expression presents an intriguing mechanism of action

In this work, we describe the identification of an auxin-regulated Ca^2+^-binding protein that functions in the root meristem and crucially regulates auxin responses and affects auxin-induced changes in cytoplasmic Ca^2+^ levels. The expression of this protein is rapidly up-regulated by auxin, and it responds to Ca^2+^ at concentrations lower than 10^−7^ M and can therefore rapidly transduce auxin signals.

## Results

### Auxin induces a specific Ca^2+^ signal pattern in the root and auxin-regulated expression of the Ca^2+^-binding protein CMI1

**Fig 1:**
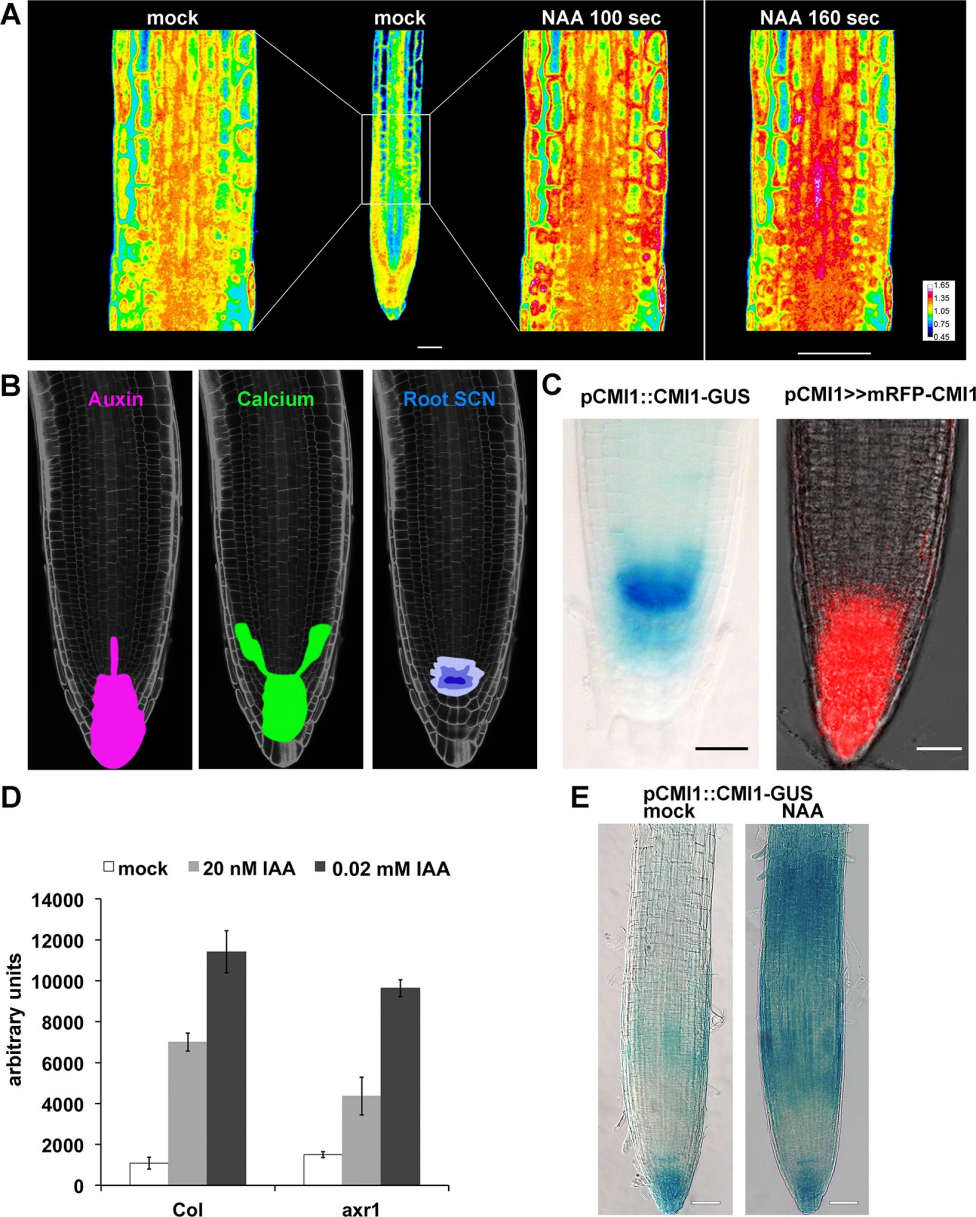
Auxin induces specific Ca2^+^ pattern and the expression of CMI1. (A) Confocal images of root expressing the YC3.6-free Ca^2+^ sensor prior to auxin treatment (mock) and after treatment with 10 μM NAA (“NAA”). The same is imaged after 100 and 160 sec of NAA treatment. (B) Images of a root with schematic representations of auxin and Ca^2+^ signal distribution at the root tip and the SCN. (C) Expression of pCMI1::CMI1-GUS in and transcription-transactivation driven pCMI1>>mRFP-CMI1 primary root meristem. (D) Microarray expression data showing the induction of *CMI1* by auxin is reduced in *axr1* auxin response mutant background. (E) Expression level and pattern of *pCMI1*-driven CMI1-GUS in *cmi1* mutant background without (mock) and 2 hours following treatment with 10 μM NAA (“NAA”). Scale bars, (A) 20 μm and (C and E) 50 μm. Underlying data for this figure can be found in S1 Data. *axr1*, auxin-resistant 1; CMI1, Ca^2+^-dependent modulator of ICR1; GUS, β-glucuronidase; IAA, indole-3-acetic acid; NAA, 1-Naphthaleneacetic acid; SCN, stem cell niche; YC3.6, Yellow Cameleon 3.6.

To study potential changes in cytoplasmic Ca^2+^ concentration in the root following auxin treatment, we used *Arabidopsis* seedlings expressing the fluorescence resonance energy transfer (FRET)-based Ca^2+^ indicator Yellow Cameleon 3.6 (YC3.6) [35]. In the control conditions, elevated Ca^2+^ concentrations were primarily observed in the root tip, resembling the auxin accumulation pattern (Fig 1A, mock and 1B and S1 Fig, mock). A typical auxin-induced Ca^2+^ signal was observed after 100 seconds of auxin (10 μM 1-Naphthaleneacetic acid [NAA]) application and increased after 160 seconds (Fig 1A, “NAA 100 sec” and “160 sec”). The most pronounced Ca^2+^ elevations were observed in the root cap, lateral root (LR) cap (LRC), and vasculature (Fig 1A, NAA and S1 Fig, NAA). The pattern of the generated Ca^2+^ signal was corresponding to auxin response and distribution ([11,36,37] and Fig 1B). The similarity between auxin-induced Ca^2+^ concentration increases (Fig 1A) and the oscillatory expression pattern of TIR1/AFB auxin receptors–regulated genes [36] is suggestive for mutual interdependency between auxin and Ca^2+^ in the root.

Previously, we identified a family of coiled-coil domain Rho of plants (ROP) effectors that we named interactors of constitutively active ROP (ICRs) [38]. Interactor of constitutively active ROP (ICR1) is degraded in an auxin-dependent fashion in the root meristem and affects root growth [38–40]. In a screen for ICR1-interacting proteins, we identified a single EF-hand Ca^2+^-binding protein that we designated as Ca^2+^-dependent modulator of ICR1 (CMI1) (*At4g27280*). CMI1 is expressed at the site of auxin accumulation, and its expression is strongly induced by auxin (Fig 1C–1E and S2 Fig). In *Arabidopsis* CMI1 is a member of a small protein family, consisting of three members, and was formerly called KIC-related protein 1 (KRP1) [41]. Because the name KRP has originally been used for the cell cycle regulators KIP-related proteins [42], which are unrelated to KRP1, we decided to adhere to the CMI1 nomenclature in this work.

### Expression of CMI1 is regulated by auxin through TIR1/AFB auxin receptors

To gain first indications for the function of CMI1 in plants, we examined the expression pattern and regulation of CMI1 expression and their correlation with cytoplasmic Ca^2+^ levels. High CMI1–β-glucuronidase (GUS) and monomeric red fluorescent protein (mRFP)-CMI1 levels were detected in the root meristem of *pCMI1*::*CMI1-GUS* and pCMI1>>mRFP-CMI1 plants (Fig 1C), resembling the expression pattern of *DR5* promoter-driven auxin reporters [4]. Microarray experiments revealed that induction of *CMI1* expression by auxin was reduced in the auxin-resistant 1 (*axr1*) auxin signaling mutant [43], indicating that auxin induces *CMI1* expression by a TIR1/AFB-dependent mechanism (Fig 1D). Furthermore, our analysis of additional publicly available microarray data revealed that *CMI1* was induced by exogenous auxin treatments and suppressed in the *axr2-1*/*iaa7* auxin-insensitive mutant [44]. A quantitative PCR (qPCR) analysis confirmed induction of CMI1 mRNA following auxin treatments (S2 Fig). In agreement, regions of increased expression of CMI1-GUS in the root elongation and maturation differentiation zones of *pCMI1*::*CMI1-GUS*/*cmi1* plants were observed following treatments with 10 μM IAA (Fig 1E), resembling the pattern of TIR/AFB auxin-induced genes [36]. Taken together, these results indicate that the expression of *CMI1* is enhanced in cells and tissues with increased auxin concentration and also regulated by auxin via the TIR1/AFB auxin receptor system.

### CMI1 mediates auxin responses and fine-tunes root growth

**Fig 2:**
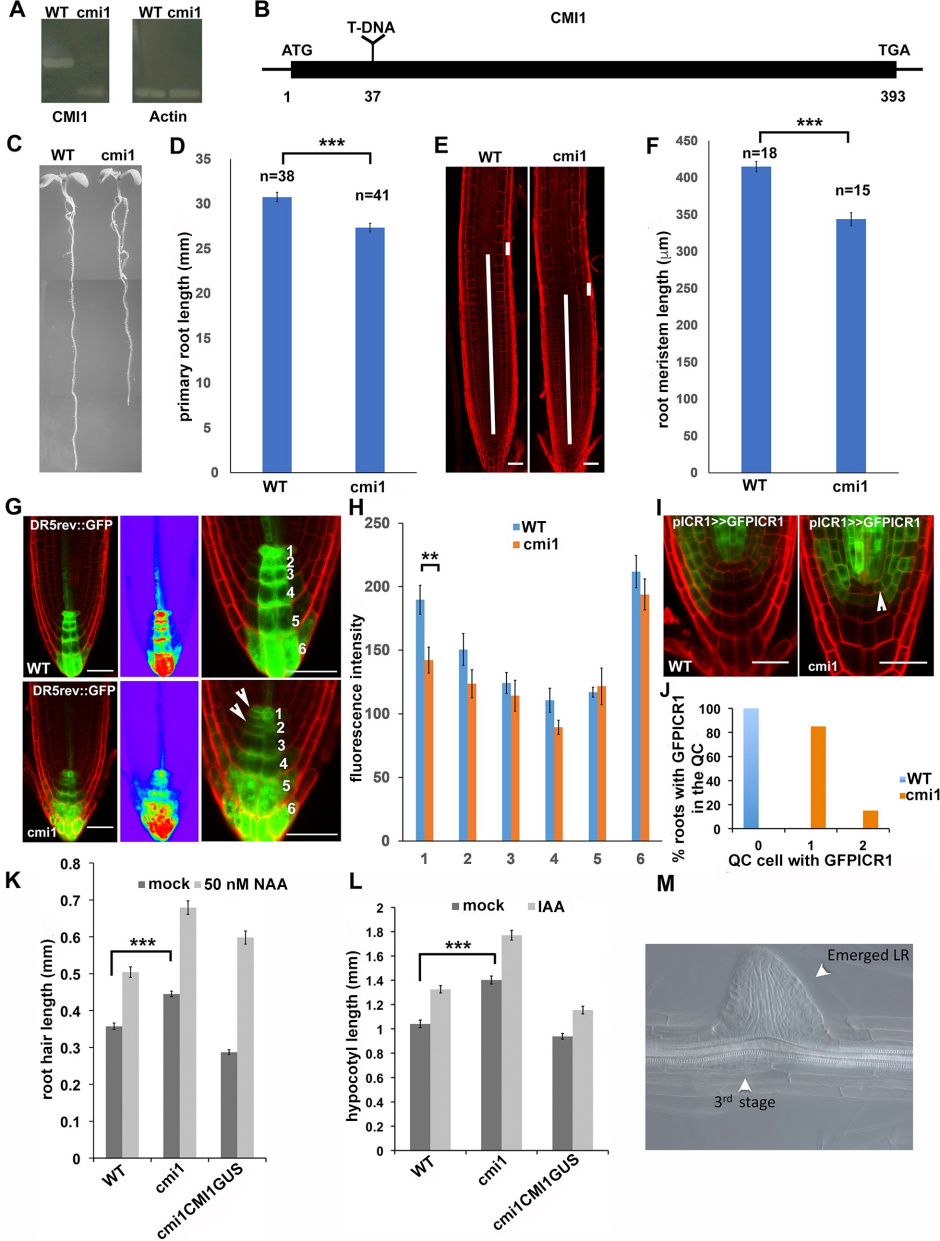
*cmi1* mutant plants have higher ICR1 levels in the QC, shorter primary root, and longer root hairs. (A) The *CMI1* RNA cannot be amplified in *cmi1*, indicating that the mutant is a null. (B) A diagram of the *CMI1* gene highlighting the T-DNA insertion at position 37. (C) *cmi1* seedlings (7 days old) have shorter primary roots. (D) Quantification of the root length in *WT* (*Ler*) and *cmi1* plants. Error bars are SE, *p* ≤ 0.001 (*t* test). (E) Root cell division zones of WT (*Ler*) and *cmi1* 7-day-old seedlings. The long bars highlight the measured root zone length. The short bars show the cell length used to determine the end of the cell division zone. (F) Quantification of the root cell division zone length calculated with root samples as shown in (E). Error bars are SE, *p* ≤ 0.001 (*t* test). (G and H) *DR5*_*rev*_::GFP auxin response maximum is reduced in *cmi1* QC. (G) Cell walls were stained with PI. The middle panels show heat diagram of the roots shown in the left panels. Right panels show higher magnifications used for quantifications. The numbers correspond to cell layers. Arrowheads highlight the signal reduction in *cmi1* compared to WT. (H) Quantification of *DR5*_*rev*_::GFP fluorescence intensity in cell layers 1–6 as defined in panel G. Layer 1 is the QC. Error bars are SE, *p* ≤ 0.01 (*t* test). (I) GFP-ICR1 expression is up-regulated in the QC (arrowhead) in *cmi1* roots. (J) Percentage of WT and *cmi1* roots with GFP-ICR1 expression in 1 or 2 QC cells. (K) Root hair length in *Ler* (WT), *cmi1*, and *cmi1* complemented with CMI1-GUS (cmi1CMI1GUS) in control (mock) or following treatments with 50 nM NAA. The root hairs in *cmi1* mutants are significantly longer than in the WT and roots complemented with CMI1-GUS. Bars are SE, *p* ≤ 0.001 (*t* test). (L) Hypocotyl length is increased in *cmi1* mutants and in response to 5 μM IAA treatments. Hypocotyls of *cmi1* mutants are significantly longer than those of the WT and seedlings complemented with CMI1-GUS. Bars are SE, *p* ≤ 0.001 (*t* test). (M) A stage 3 LRI developing opposite to an emerging LRI in a *cmi1* root. Scale bars, (E) 50 μm and (G and I) 20 μm. Underlying data for this figure can be found in S1 Data. CMI1, Ca^2+^-dependent modulator of ICR1; GFP, green fluorescent protein; GUS, GUS, β-glucuronidase; ICR1, interactor of constitutively active ROP; *Ler*, Landsburg *erecta*; LR, lateral root; LRI, LR initial; PI, propidium iodide; QC, quiescent center; WT, wild type.

Next, we further examined the function of CMI1 and its interconnection with auxin signaling in plants by analyzing the phenotype of a *CMI1* loss-of-function mutant. The *cmi1* mutant (Cold Spring Harbor Laboratory [CSHL] line GT_24505) carries a transposon insertion at nucleotide 37 in the *CMI1* coding region (Fig 2A and 2B). Compared to a wild-type (WT) control, the *cmi1* plants have shorter primary roots (Fig 2C and 2D) as a result of a smaller root meristem size, defined as the length of the region between the QC and the initiation of the elongation zone (Fig 2E and 2F). Importantly, the shorter primary root phenotype was complemented by *pCMI1*::*CMI1-GUS* (S3A Fig), confirming that the mutant phenotype resulted from the loss of *CMI1* function and that the observed expression pattern of *pCMI1*::*CMI1-GUS* reflects the expression pattern of the endogenous *CMI1* gene.

Next, we determined whether the loss of *CMI1* function affects auxin response using the *DR5*_*rev*_::*GFP* auxin response marker [45,46]. In *cmi1* mutant roots, the auxin response maximum in the QC was reduced, compared to a WT control (Fig 2G). Quantification of the green fluorescent protein (GFP) fluorescence levels revealed a significant reduction (*p* ≤ 0.006, *t* test) in fluorescence level in the QC cells (Fig 2H). Ectopic expression of GFP-ICR1 was detected in the QC cells of the *cmi1* mutant but not in WT roots (Fig 2I and 2J), in line with the reduced auxin response in the QC [39,40]. Hence, CMI1 affects ICR1 levels, indirectly by regulating the auxin response.

The regulation of *CMI1* expression by auxin prompted us to examine the possible involvement of CMI1 in further well-characterized auxin responses. The initiation and elongation of root hairs are regulated by TIR1/AFB-Aux/IAA-dependent auxin signaling [47–49]. We found that root hairs were longer in the *cmi1* mutant compared to WT and *cmi1/pCMI1*::*CMI1GUS* plants and were elongated in response to exogenous auxin treatments (Fig 2K and S3C Fig). TIR1/AFB-Aux/IAA-dependent auxin signaling also affects hypocotyl length [50,51]. Hypocotyls of the *cmi1* mutant were significantly longer (*p* ≤ 0.001, *t* test), compared to WT and *cmi1/pCMI1*::*CMI1GUS* plants. As expected, external IAA treatments induced hypocotyl elongation in WT, *cmi1* mutant, and *cmi1/pCMI1*::*CMI1 GUS* plants (Fig 2L and S3B Fig). LR formation is regulated by both auxin response and distribution [45,52–55]. *cmi1* plants exhibited abnormal LR patterning (Fig 2M and S4 Fig), with an average of 7 LRs/cm in *cmi1* compared to 4 LRs/cm in control Landsburg *erecta* (*Ler*) seedlings. Together, the changes in *DR5*_*rev*_::GFP and GFP-ICR1 expression pattern and the macroscopic phenotype of *cmi1* mutant plants suggest that CMI1 regulates both the spatial distribution and the level of auxin responses.

**Fig 3:**
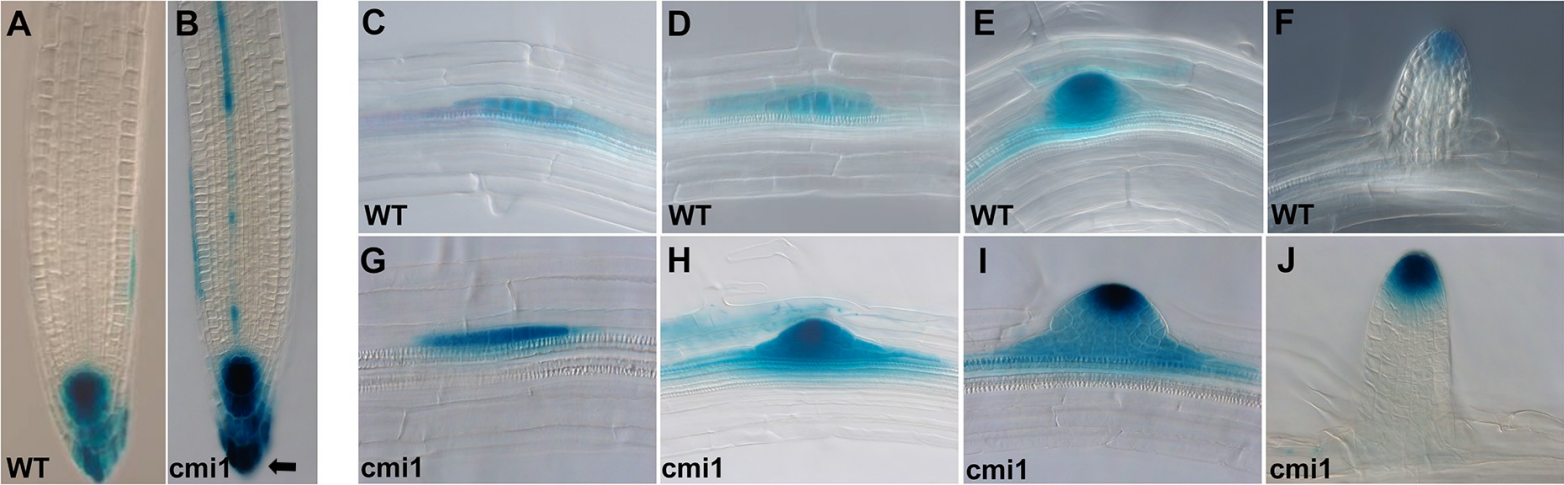
CMI1 loss of function results in enhanced auxin-induced *DR5*::GUS expression. Expression level of *DR5*::*GUS* auxin response marker in roots of *Ler* (WT) and *cmi1* (B). Note the signal shift toward the columella in *cmi1/DR5*::*GUS* (arrow). (C-J) Expression levels of *DR5*::*GUS* in LRI of *Ler* (WT) (C-F) and *cmi1* (G-J). CMI1, Ca^2+^-dependent modulator of ICR1; *Ler*, Landsburg *erecta*; LRI, lateral root initial; WT, wild type.

Corresponding to the increased auxin response of *cmi1* mutants, the *DR5*::*GUS* staining was stronger in *cmi1* primary root and LR initials compared to WT control (Fig 3A–3J). Notably, similar to the results with the *DR5*_*rev*_::GFP, a shift in the *DR5*::GUS signal toward the columella in *cmi1*/DR5::GUS seedlings was also observed (Fig 3B, arrow).

To examine a potential impact of CMI1 function on the regulation of auxin transport and PIN polarity, we carried out whole-mount immunostaining of roots with anti-PIN1 and anti-PIN2 antibodies. Importantly, these immunostaining experiments revealed that PIN2 distribution in the cortex is altered in *cmi1* (S5A Fig). In 55% of the cells, PIN2 displayed apical localization, and in another 25%, it was nonpolar. In comparison, in *Ler* (WT) in 90% of the cells, PIN2 displayed basal localization in the cortex, and in only 10% of the cells, it was either apical or nonpolar (S5B Fig).

### CMI1 affects auxin-induced Ca^2+^ signaling in a cell type/tissue–specific manner

**Fig 4:**
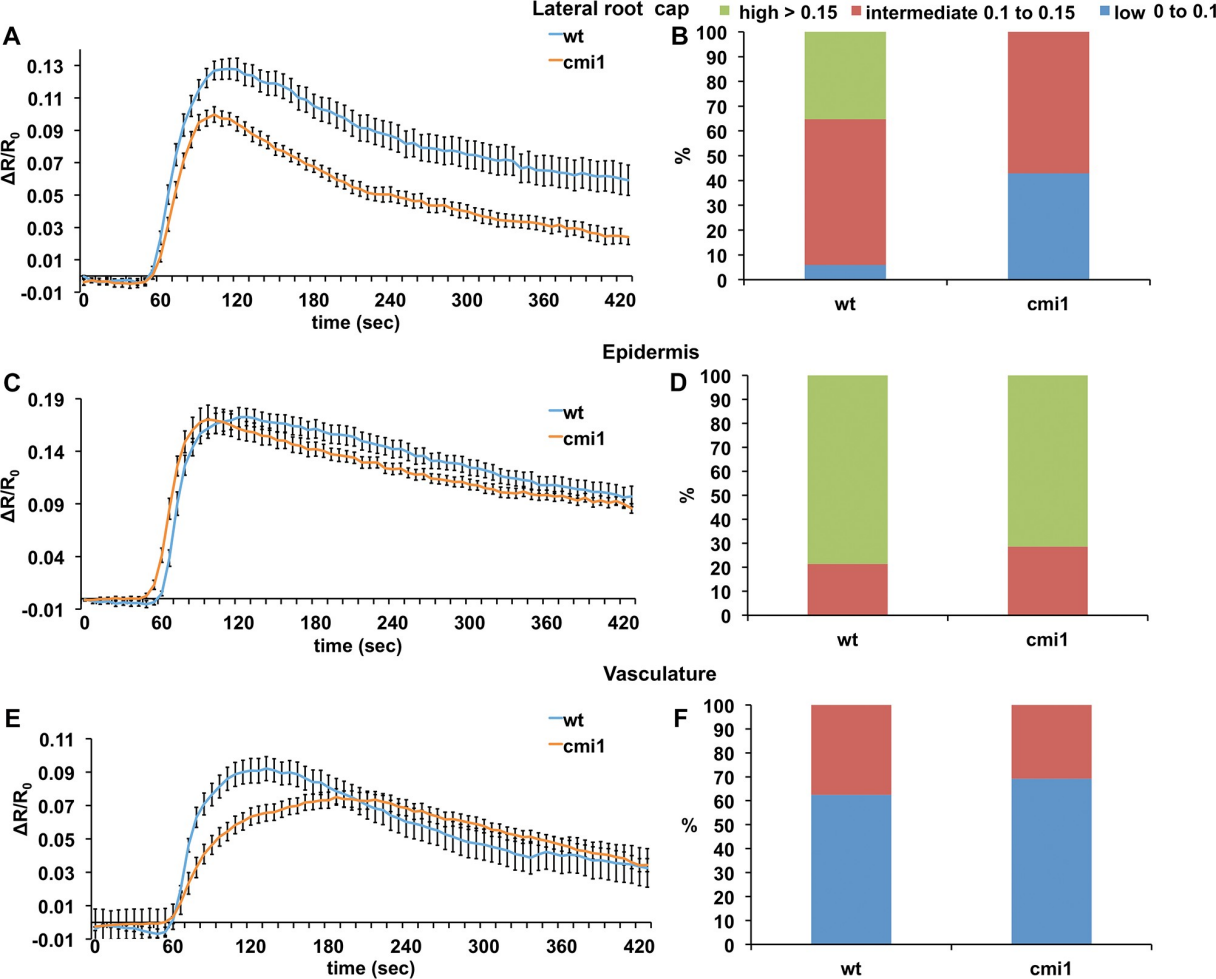
Auxin-induced Ca^2+^ responses are reduced in *cmi1* in a tissue-specific fashion. Auxin-induced Ca^2+^ responses in lateral root cap (A and B), epidermis (C and D), and vascular tissues (E and F). Note the reduced Ca^2+^ levels and different kinetics in Ca^2+^ decrease and increase in the lateral root cap and the vascular tissues, respectively. Error bars are SE. Underlying data for this figure can be found in S1 Data. *cmi1*, Ca^2+^-dependent modulator of ICR1; wt, wild type.

To further examine a potential role of CMI1 in interconnecting Ca^2+^ signaling and auxin function possibility, we compared the auxin-induced cytoplasmic Ca^2+^ signals in the LRC, epidermis, and vasculature of WT and *cmi1* roots expressing YC3.6. The quantification of Ca^2+^ dynamics was carried out by calculating the ratio change in the FRET signals (ΔR/R_0_) (Fig 4A–4F). Quantitative analyses revealed genotype-specific differences in the tissue specificity and intensity of auxin-induced cytoplasmic Ca^2+^ signals between *Ler* (WT) and *cmi1*. In the LRC, the auxin-induced cytoplasmic Ca^2+^ response displayed a higher amplitude in the WT *Ler* than in *cmi1* mutants. Specifically, none of the *cmi1* roots, as opposed to 35% of the analyzed WT roots, showed a ratio change ΔR/R_0_ ≥ 0.15. In contrast, 40% of the *cmi1* roots displayed lower Ca^2+^ elevations, characterized by ΔR/R_0_ ranging between 0 and 0.1, whereas only 5% of the WT roots displayed such low Ca^2+^ signals in the LRC (Fig 4A and 4B). In the epidermis, strong increases in Ca^2+^ levels (ΔR/R_0_ ≥ 0.15) predominated in both the WT and *cmi1* backgrounds, with only slightly fewer samples with high Ca^2+^ levels detected in *cmi1* mutants (Fig 4C and 4D). In the vasculature, the amplitude of the auxin-induced Ca^2+^ signals was comparable in WT and *cmi1* mutant. The low-threshold Ca^2+^ signals (ΔR/R_0_ ranging between 0 and 0.1) predominated in both WT and *cmi1* backgrounds, and only 10% more WT roots displayed higher Ca^2+^ levels, with ΔR/R_0_ values ranging between 0.1 and 0.15 (Fig 4E and 4F). However, there was a striking difference in the shape of the signal and in the kinetics of the signal to reach the maximum amplitude. The WT roots evoked a maximum Ca^2+^ response in 120 seconds, whereas in the *cmi1* mutants, the Ca^2+^ maxima were reached in 230 seconds. Interestingly, in the vasculature, the restoration of basal level of Ca^2+^ followed a similar kinetics. Taken together, these results reveal that loss of *CMI1* function alters auxin-induced Ca^2+^ signals, especially in the LRC and vascular cells, and suggest that CMI1 regulates auxin-associated changes in cytoplasmic Ca^2+^ levels in a cell/tissue-specific fashion. Moreover, our finding points to an elaborate cell specificity and diversity in complex tissues.

**Fig 5:**
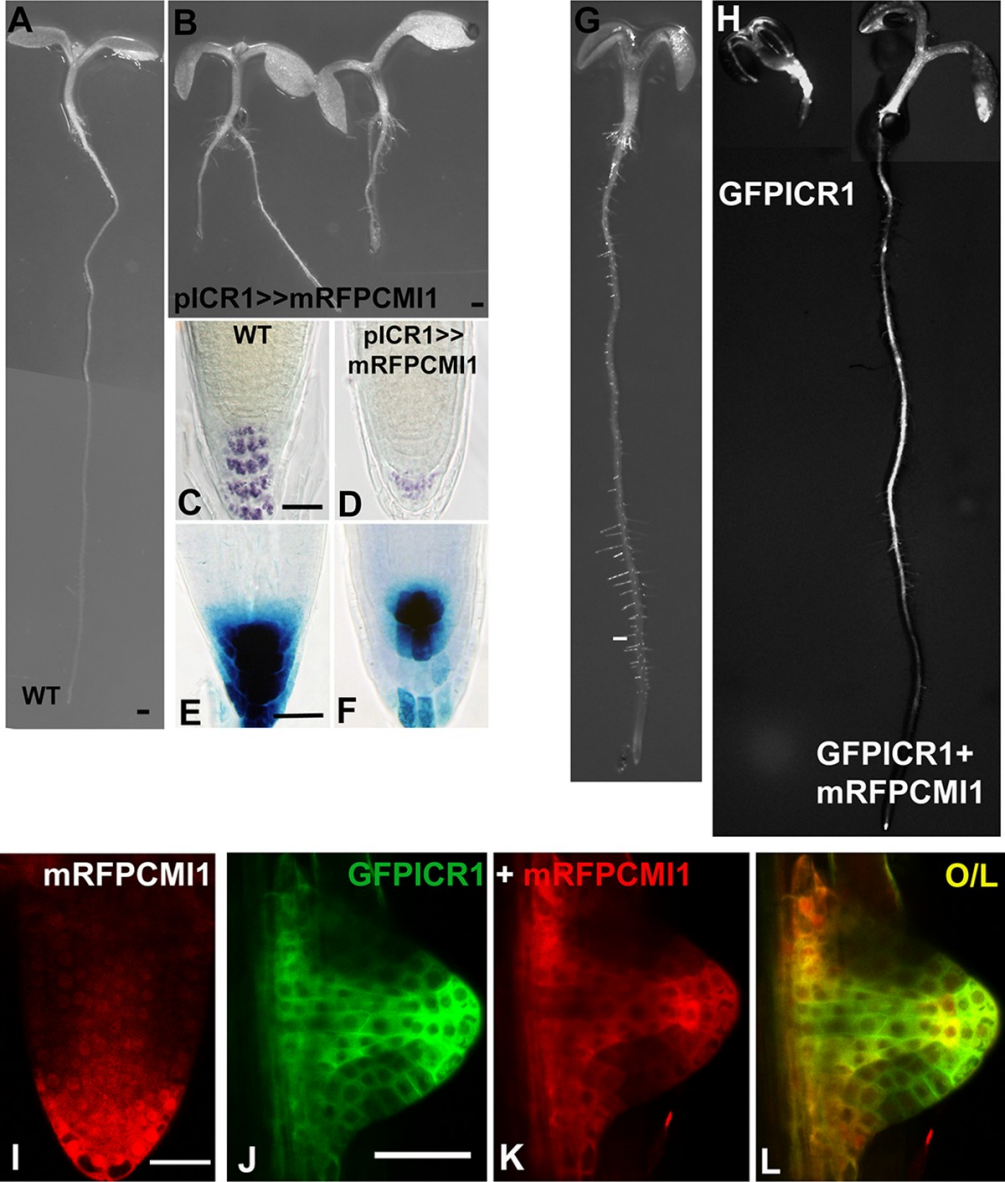
Ectopic expression of CMI1 suppresses root development and auxin response. (A) Control *Col-0* (WT) seedling. (B) Root growth is arrested in *pICR1>>mRFP-CMI1* seedlings. (C and D) Reduced iodine (IKI) columella staining in a *pICR1>>mRFP-CMI1* root. (E and F) Reduced auxin response in a *pICR1>>mRFP-CMI1 DR5*::*GUS* root. (G) A control *pCMI2>>LhG4* plant. (H) Root development is inhibited in a *pCMI2>>GFP-ICR1* plant (left) and restored by coexpression of GFP-ICR1 and mRFP-CMI1 in *pCMI2>>GFP-ICR1/mRFP-CMI1* plants (right). (I) mRFP-CMI1 is expressed in the lateral root meristem QC and initial cells and accumulates in the cytoplasm and nuclei in *pCMI1>>mRFP-CMI1* plants. (J-L) GFP-ICR1 and mRFP-CMI1 are colocalized in the cytoplasm in a pCMI2>>GFP-ICR1/mRFP-CMI1 lateral root initial. Note the absence of mRFP-CMI1 from nuclei. Scale bars 0.5 mm (A, B, G and H), 50 μm (C-F), and 50 μm (I-L). CMI1, Ca^2+^-dependent modulator of ICR1; *Col-0*, *Arabidopsis Columbia-0*; GFP, green fluorescent protein; ICR1, interactor of constitutively active ROP; mRFP, monomeric red fluorescent protein; QC, quiescent center; WT, wild type.

To further examine the function of CMI1, we ectopically expressed mRFP-CMI1 under regulation of the ICR1 promoter (*pICR1>>mRFPCMI1*), using a transcription-transactivation system [56]. The roots of *pICR1>>mRFPCMI1* plants were short and had reduced columella layers, indicating root meristem collapse, and reduced auxin response maxima (Fig 5A–5F). Hence, ectopic expression of CMI1 was associated with reduction in auxin levels or responses and suppression of root growth.

Previously, we demonstrated that inducing elevated levels of ICR1 in the QC by its expression under regulation of the CMI2 (At5g54490) promoter, utilizing the pOp/LhG4 transcription/transactivation system (pCMI2>>GFP-ICR1), resulted in inhibition of root growth [40] and (Fig 5G and 5H). Remarkably, coexpression of GFP-ICR1 and mRFP-CMI1 in *pCMI2>>GFP-ICR1/mRFP-CMI1* resulted in suppression of root growth arrest (Fig 5H). In LR primordium, mRFP-CMI1 was detected in nuclei, cytoplasm, and plasma membrane (Fig 5I), similar to its distribution in the leaf epidermis pavement cells (S6A Fig) and in agreement with the protein immunoblot with anti-CMI1 antibodies that indicated localization in both soluble and insoluble fractions (S6B Fig). Examination of the subcellular localization of both GFP-ICR1 and mRFP-CMI1 revealed that when coexpressed together with GFP-ICR1 in the same cells, mRFP-CMI1 was excluded from nuclei (Fig 5J–5L). These data indicated that coexpression of CMI1 and ICR1 in the same cells affected the subcellular distribution of CMI1 and its function.

### Ca^2+^ promotes the interaction between CMI1 and ICR1

**Fig 6:**
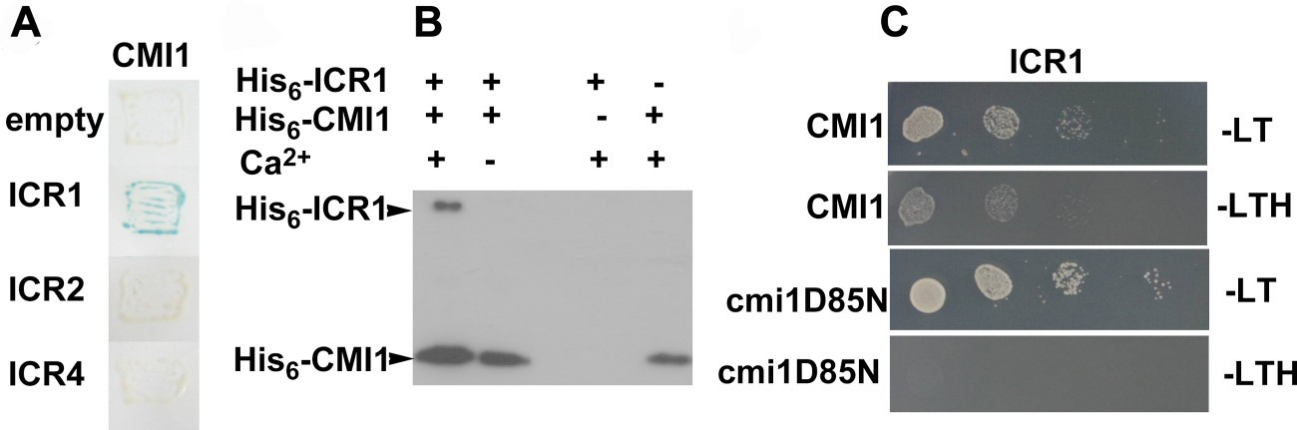
CMI1 specifically interacts with ICR1 in a Ca^2+^-dependent fashion. (A) CMI1 interacts with ICR1 but not with ICR2 or ICR4 in yeast two-hybrid assays. (B) Protein immunoblot decorated with anti polyHis-tag monoclonal antibodies showing that coimmunoprecipitation of His-CMI1 and His-ICR1 is Ca^2+^-dependent. (C) ICR1 interacts with CMI1 but not with the cmi1D85N Ca^2+^ nonbinding mutant in yeast two-hybrid assays. CMI1, Ca^2+^-dependent modulator of ICR1; ICR, interactor of constitutively active ROP; -LT, Leu-, Trp-deficient medium; -LTH, Leu-, Trp-, His-deficient medium.

CMI1 is a small 14-kDa protein containing a single EF-hand (S7A Fig). To gain the molecular mechanism of CMI1 action, we characterized its interaction with ICR1 and whether it is Ca^2+^-dependent. CMI1 interacted specifically with ICR1 but not with ICR2 (*At2g37080*) or ICR4 (*At1g78430*), the closest homologues of ICR1 (Fig 6A). To further characterize the interaction between CMI1 and ICR1 and to examine whether it is Ca^2+^-dependent, we performed in vitro pull-down experiments using either anti-CMI1 antibodies to examine whether they coimmunoprecipitate His-ICR1 with His-CMI1 or glutathione S-transferase (GST)-ICR1 to examine whether, when bound to glutathione sepharose beads, it would precipitate His-CMI1. His-ICR1 was immunoprecipitated together with His-CMI1 using anti-CMI1 antibodies in the presence of Ca^2+^ (Fig 6B). In contrast, pull-down of His-CMI1 by GST-ICR1 did not take place when the Ca^2+^ was chelated with EGTA. The interaction of ICR1 and CMI1 in the pull-down assays was specific, since His-CMI1 was not precipitated by nonfused GST or glutathione beads (S7B and S7C Fig). To further corroborate that the interaction between ICR1 and CMI1 is Ca^2+^-dependent, we created a CMI1 D85N mutant in which a conserved EF-hand Asp required for Ca^2+^ binding [57] was mutated to Asn (S7A Fig). Yeast two-hybrid assays showed that CMI1 interacts with ICR1 but not with the cmi1D85N protein (Fig 6C). Taken together, these results establish that the interaction between ICR1 and CMI1 is Ca^2+^-dependent both in yeast and in vitro.

### CMI1 binds Ca^2+^ at low concentrations and functions as a monomer

**Fig 7:**
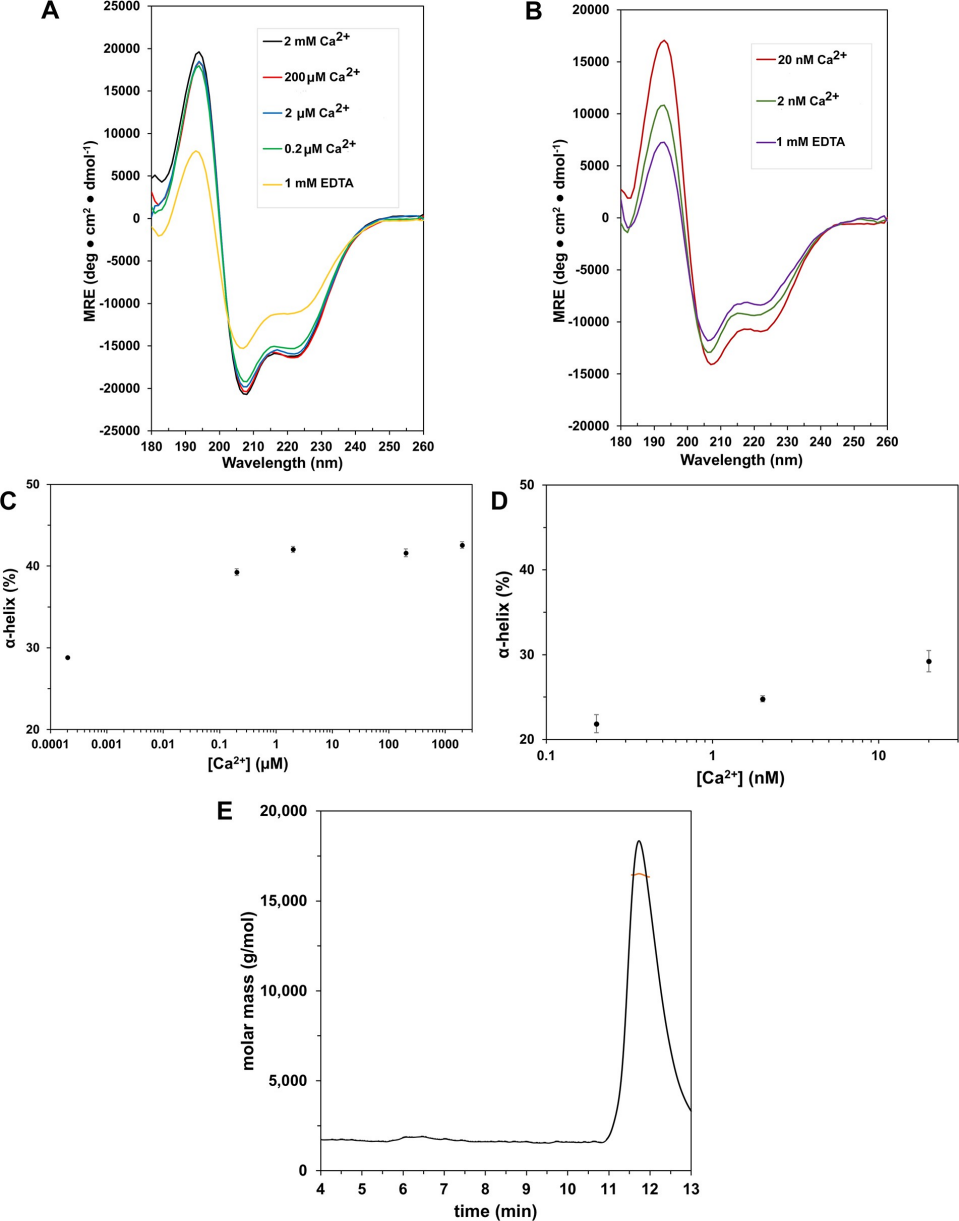
CMI1 changes secondary structure in free Ca^2+^ concentration ranging between 10^−9^ and 10^−8^ M and is a monomer in solution. (A and B) CD spectra of 60 μM CMI1 at indicated free Ca^2+^ concentrations. Each curve is labeled as per legends. Measurements presented in (A and B) were carried out on different days. (C and D) Percentage of α-helix of CMI1 at different free Ca^2+^ concentrations calculated from the CD spectra in (A and B), respectively. (E) An SEC-MALS elution profile of 4 μg CMI1 in 2 mM Ca^2+^ solution. CMI1 eluted as a single peak with a molecular mass (red line) corresponding to a monomeric form. Underlying data for this figure can be found in S1 Data. CD, circular dichroism; CMI1, Ca^2+^-dependent modulator of ICR1; ICR, interactor of constitutively active ROP; M, million; MRE, mean residue ellipticity; SEC-MALS, size-exclusion chromatography multiangle light scattering.

Circular dichroism spectroscopy (CD-spec) has previously been used to monitor changes in secondary structure of Ca^2+^-binding proteins and, by that, assess the Ca^2+^ binding [58–63]. Here, CD-spec was used to examine changes in CMI1 secondary structure at different free Ca^2+^ concentrations. The analysis was carried out in solutions with the following free Ca^2+^ concentrations: 10^−10^ M Ca^2+^ (1 mM EDTA), 2 nM, 20 nM, 0.2 μM, 2 μM, 200 μM, and 2 mM Ca^2+^. Because of technical limitations, the measurements at free Ca^2+^ concentrations of 2 nM and 20 nM and 0.2 μM, 2 μM, 200 μM, and 2 mM were carried out on different days. Control measurements in 1 mM EDTA solutions were carried out on both days (Fig 7A and 7B). The CMI1 CD spectra at free Ca^2+^ concentrations ranging between 0.2 μM to 2 mM were similar but were all significantly different from the 1 mM EDTA Ca^2+^-free solution (Fig 7A). The CD spectra of CMI1 in 20 nM free Ca^2+^ concentrations were also significantly different from the Ca^2+^-free 1 mM EDTA solution, and also, different spectra were observed at 2 nM free Ca^2+^ (Fig 7B). The percentage of α-helices that were calculated based on the CD spectra were around 40% for free Ca^2+^ concentrations ranging between 0.2 μM and 2 mM and below 30% for CMI1 in the Ca^2+^-free 1 mM EDTA solution (Fig 7C). Although the percentage of α-helices (Fig 7D) were lower compared to the measurements presented in Fig 7C, the differences in α-helix content between the 20 nM free Ca^2+^ and 1 mM EDTA were around 10%, similar to the differences between the 0.2 μM–2 mM Ca^2+^ and the Ca^2+^-free 1 mM EDTA solutions (compare Fig 7C and 7D). The CD-spec analysis suggested that CMI1 can bind Ca^2+^ at free Ca^2+^ concentrations ranging between 10^−9^ and 10^−8^ M, which in turn induce secondary structure changes that result in an increase in α-helicity. Steady-state cytoplasmic Ca^2+^ concentrations [Ca^2+^]_cyt_ have been estimated to be around 100 nM [64,65]. Similar values of 50–90 nM of resting [Ca^2+^]_cyt_ along the root have recently been reported [66]. Thus, it appears well conceivable that CMI1 is bound to Ca^2+^ and functional also at steady-state [Ca^2+^]_cyt_. Furthermore, since CMI1 expression is up-regulated by auxin and highly expressed when auxin response and Ca^2+^ levels are high (Fig 1), it likely functions as an auxin-dependent signaling transducer.

Many Ca^2+^-binding proteins require at least two EF-hands for their function or function as dimers if they contain a non-even number of EF-hands. We therefore hypothesized that CMI1, bearing only a single Ca^2+^-binding EF-hand, might oligomerize in solution. Therefore, the quaternary structure of CMI1 was examined by size-exclusion chromatography multiangle light scattering (SEC-MALS). To eliminate potential effects of the poly-His-tag on solution structure, we performed the analysis on recombinant bacterially expressed and purified recombinant CMI1 from which the poly-His-tag was cleaved. At both concentrations of 2 and 4 mg/ml, CMI1 eluted as a monodisperse species at around 11.5 minutes with a measured molecular mass of 16 kDa, corresponding to the monomeric form of the protein (Fig 7E and S8 Fig). Hence, we conclude that CMI1 is strictly monomeric in vitro at least to concentrations of 25 μM in high Ca^2+^ conditions. This finding, however, does not exclude that CMI1 upon interaction with additional proteins may form oligomeric assemblies.

Possible homo- and heterodimerization of CMI1 was also examined by yeast two-hybrid assays. The analysis was carried out with Clontech LexA yeast two-hybrid yeast strain *EGY48*, since CMI1 activates gene expression in Gal4-based yeast two-hybrid strains when expressed fused to Gal4 DNA-binding domain (Gal4-DB). Following 24 hours of incubation, a very faint blue color appeared in X-Gal assays of CMI1-LexA-BD/CMI1-Lex-activation domain (AD) compared to a strong blue color in the CMI1-ICR1 and no color in the vector-control assays. Following 48 hours of incubation, the X-Gal assays of the CMI1-BD/CMI-AD assays had a light blue color compared to the strong blue of the CMI1-ICR1 and no color in the negative vector-control assays (S9 Fig). Together, the yeast two-hybrid assays suggest that CMI1 could form dimers in yeast in the absence of ICR1 but also that the high affinity to ICR1 would interfere with this homodimerization. Therefore, the differences in the strength of the interaction in yeast and the SEC-MALS results strongly suggest that CMI1 very likely interacts with ICR1 as a monomer.

### Interaction of CMI1 with ICR1 involves a conserved hydrophobic pocket in CMI1 and a CaM binding-like domain (CBLD) in ICR1

**Fig 8:**
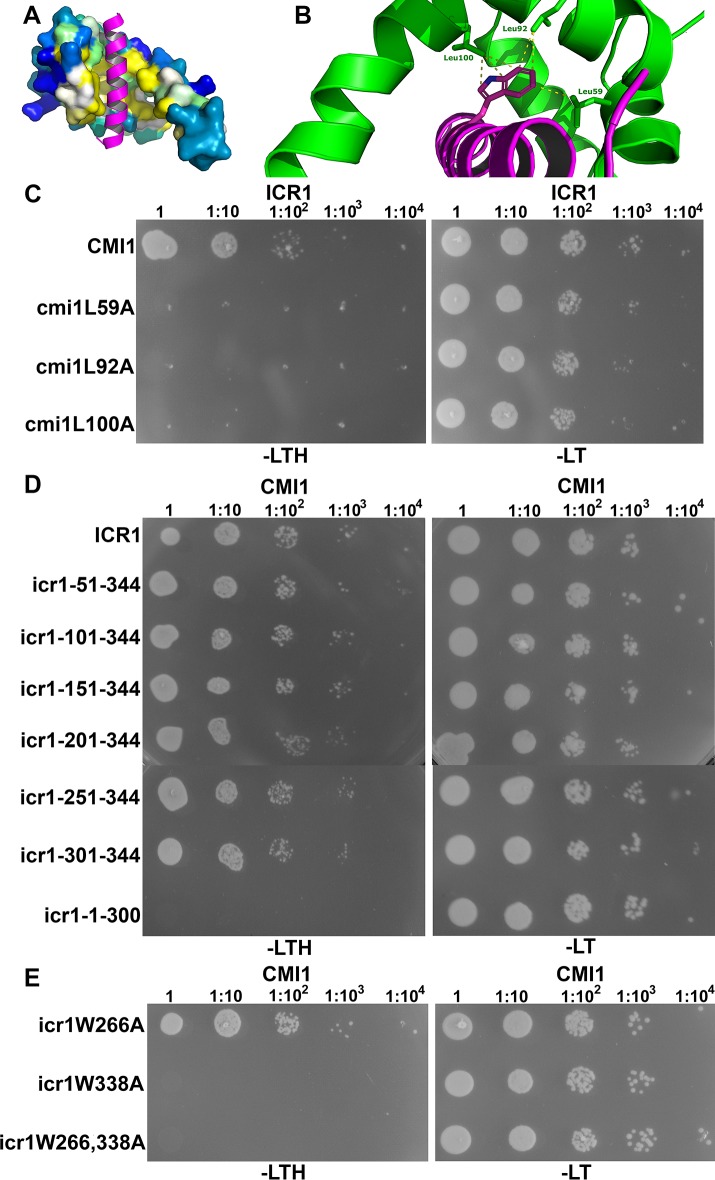
Interaction between CMI1 and ICR1 requires a hydrophobic pocket in CMI1 and a C-terminal W338 residue in ICR1. (A and B) A homology model of CMI1 with the CBD of KCBP shown in magenta. (A) A surface representation of CMI1 with residues of the hydrophobic pocket highlighted in yellow. (B) A close-up displaying CMI1 Leu residues L59, 92, and 100 (green) interacting with a Trp residue in KCBP CBD (magenta). (C-E) Yeast two-hybrid assays. (C) ICR1 did not interact with CMI1 hydrophobic pocket L59, L92, and L100 mutants. (D) ICR1 44 C-terminal residues are required and sufficient for interaction with CMI1, but interactions are detected also at 1:10^4^ dilution with icr1-151-344 C-terminal or longer fragments. (E) ICR1 Trp residue W338 but not W266 is required for the interaction between CMI1 and ICR1. (C-E) Numbers above panels denote dilutions of the yeast cells. CBD, calmodulin-binding domain; CMI1, Ca^2+^-dependent modulator of ICR1; ICR, interactor of constitutively active ROP; KCBP, kinesin-like CaM-binding protein; -LT, Leu-, Trp-deficient medium; -LTH, Leu-, Trp-, His-deficient medium.

Having established that CMI1 could function as a Ca^2+^ sensor, we sought to obtain more insights into the molecular details of its structure and function. The 3D structure of CMI1 was predicted using homology modeling based on the structure of KIC, which belongs to the same subfamily single EF-hand Ca^2+^-binding proteins [41,67]. The predicted structure of CMI1 suggests the formation of two helix-loop-helix domains, one which binds Ca^2+^ and one which does not. This structural feature likely enables CMI1 to function as a monomer with regard to Ca^2+^ binding (Fig 8A and 8B). The CMI1 structure with Ca^2+^ bound is predicted to form a hydrophobic pocket (Fig 8A, residues highlighted in yellow). Modeling of CMI1 in complex with the CaM-binding domain (CBD) of the KIC interactor kinesin-like CaM-binding protein (KCBP) [41,67] revealed that three Leu residues in the putative hydrophobic pocket of CMI1—namely, L59, L92, and L100—can potentially serve as interacting side chains with a Trp residue in a domain that would be structurally related to a CBD, which we therefore designated as CBLD (Fig 8B).

To test the hypothesis that L59, L92, and L100 form a hydrophobic pocket, we exchanged L to A in each of the respective Leu residues and tested the interaction of this modified CMI1 versions with ICR1 in yeast. As expected, neither cmi1 mutants L59A nor L92A nor L100A interacted with ICR1 in yeast two-hybrid assays (Fig 8C), strongly suggesting that the three Leu residues are part of a hydrophobic pocket required for protein–protein interaction. To ensure that the mutations do not cause structural modifications in the hydrophobic pocket, an aligned structural model of CMI1 and its hydrophobic pocket Leu mutants was created. This model confirmed that the mutations have not caused structural changes within the core CMI1 that contains the hydrophobic pocket, confirming the requirement for the Leu residues (S10 Fig). It is very likely that the structural differences that are observed in the N- and C-terminal domains result from the low confidence of the model in these regions.

There are two Trp residues in the C-terminal end of ICR1 at positions 266 and 338 that could be part of a potential CBLD. To map a potential CMI1-interaction domain in ICR1, we generated a series of N- and C-terminal deletion mutants of ICR1 and examined their interaction with CMI1 in yeast two-hybrid assays. These analyses revealed that the 44 C-terminal residues of ICR1 (icr1-301-344) are necessary and sufficient for interaction with CMI1 (Fig 8D). Slightly stronger yeast growth, which was apparent at dilutions of 1:10^3^ and 1:10^4^, that resembled the full-length ICR1 was observed between an ICR1 C-terminal fragment encompassing residues 151 to 344 (icr1-151-344) and CMI1 (Fig 8D). Hence, the C-terminal 44-residue domain of ICR1 can function as a CBLD, but possibly, other residues also contribute to the interaction.

Next, we examined the interaction between CMI1 and ICR1 harboring the single amino acid substitutions W266A and W338A. In yeast two-hybrid assays, ICR1W266A still interacted with CMI1 whereas ICR1W338A did not (Fig 8E). Similar results were obtained when W266 and 338 were mutation to Gln (Q) (S11 Fig). Together, these results suggest that W338 is the primary Trp residue in ICR1 CBLD that is most crucial for interaction with residues in the hydrophobic pocket of CMI1.

**Fig 9:**
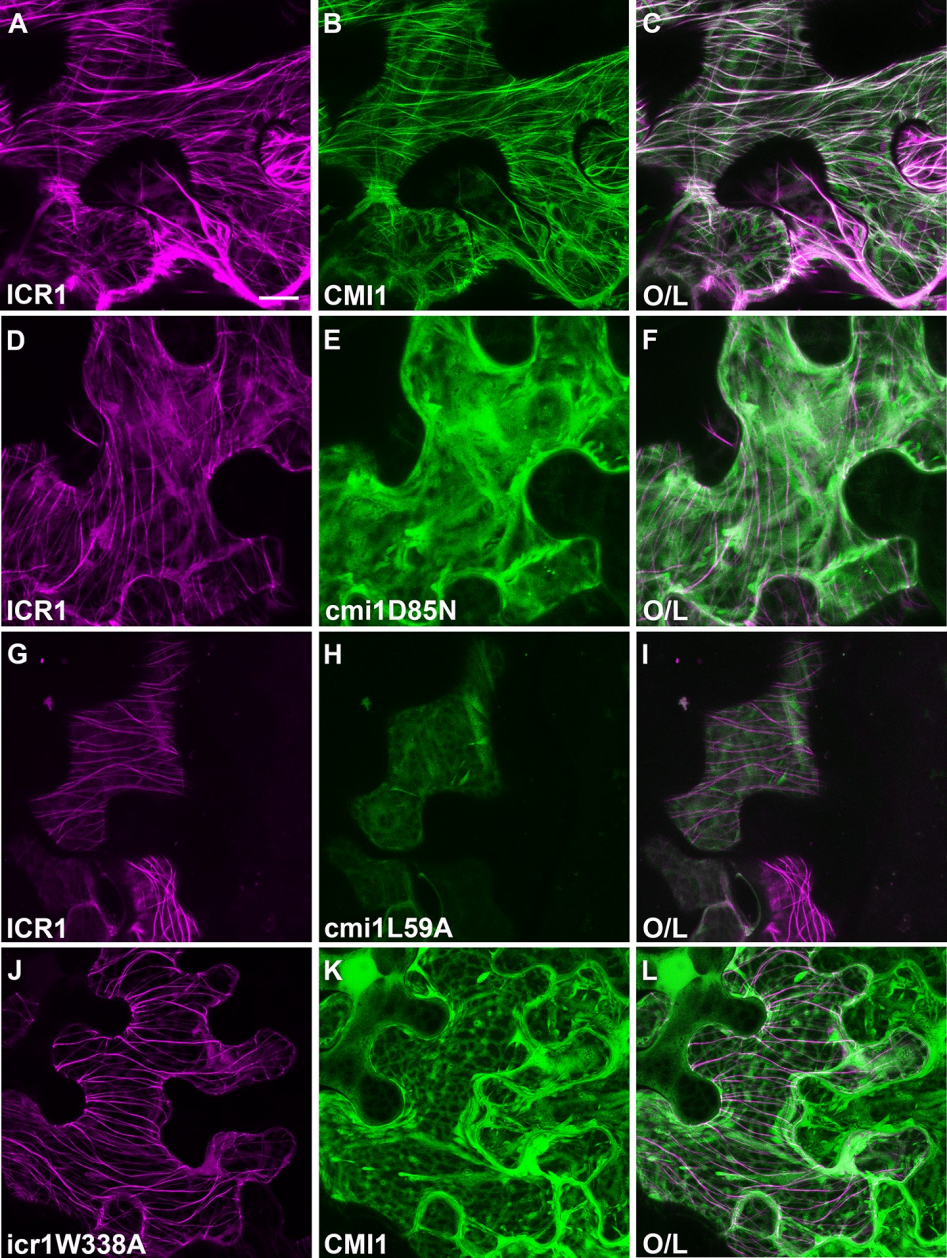
Recruitment of CMI1 by ICR1 to MTs depends on Ca^2+^ binding and intact hydrophobic pocket of CMI1 and ICR1 W338. (A-I) CMI1, but not Ca^2+^ nonbinding cmi1D85N and hydrophobic pocket cmi1L59A, mutants is recruited to MTs by ICR1. (J-L) icr1W338A is associated with MTs but does not recruit CMI1. Each panel is as per legends. Bar, 20 μm for all panels. CMI1, Ca^2+^-dependent modulator of ICR1; ICR, interactor of constitutively active ROP; MT, microtubule; O/L-overlay of mCherry and GFP signals.

Next, we examined the localization of CMI1 and ICR1 and the potential influence of interaction with ICR1 on CMI1 localization in plants. When expressed in plants, ICR1-mCherry localized to microtubules (MTs), as indicated by its colocalization with the MTs marker Tubulin alpha-6 (TUA6)-GFP (S12A–S12C Fig). When expressed by itself in *Arabidopsis* under control of its own promoter, CMI1 was observed at the plasma membrane, throughout the cytoplasm, and in nuclei (S6 Fig). Imaging of leaf epidermis pavement cells showed the mRFP-CMI1 is indeed localized to the plasma membrane as well as to nuclei and cytoplasm (S6A Fig). Furthermore, protein immunoblot with anti-CMI1 antibodies indicated that CMI1 is localized in soluble and insoluble fractions in different tissues (S6B and S6C Fig). However, when ICR1 and CMI1 were transiently coexpressed in *Nicotiana benthamiana* leaf epidermis cells, ICR1-mCherry and GFP-CMI1 were localized to MTs (Fig 9A–9C). The colocalization of both mCherry-ICR1 and GFP-CMI1 was sensitive to the anti-MT drug oryzalin, confirming that they were both localized to MTs (S12D–S12F Fig). In contrast to GFP-CMI1, neither GFP-CMI1D85N (mutated in the Ca^2+^ binding EF-hand) nor GFP-CMI1L59A (mutated in the hydrophobic pocket) were recruited to MTs (Fig 9D–9F and 9G–9I). Likewise, when GFP-CMI1 was coexpressed with ICR1W338A-mCherry, it was not recruited to MTs, although ICR1W338A-mCherry was observed on MTs (Fig 9J–9L). Taken together, the coexpression assays in plants reinforced the combined conclusions derived from the results of the structural modeling and interaction assays, demonstrating that also in plant cells, the interaction between ICR1 and CMI1 is very likely Ca^2+^-dependent and involves a hydrophobic pocket in CMI1 and a C-terminal CBLD involving W338 in ICR1. These results also provide the possibility that CMI1 modulates the function of ICR1 and/or fulfills alternative functions in its ICR-bound and non–ICR-bound form.

## Discussion

### CMI1 function in auxin-regulated Ca2+ response

The specificity of Ca^2+^ response has been proposed to be regulated by the differential response of Ca^2+^ sensors to fluctuations in cellular Ca^2+^ levels, referred to as the Ca^2+^ code/language, and the specificity of Ca^2+^ sensors toward specific targets [33,68]. A third regulatory mechanism is the expression regulation of Ca^2+^-binding proteins in time and space. In the root, the uneven distribution of auxin regulates cell-specific gene expression patterns [36]. Hence, auxin-regulated Ca^2+^ binding proteins are candidates for carrying out cell-specific responses. CMI1, whose expression is regulated directly through TIR1/AFB-Aux/IAA auxin receptors–coreceptors, is an example of Ca^2+^-binding protein whose cell-specific expression is regulated by auxin (Fig 1). Results of this work also indicate that CMI1 can bind Ca^2+^ at free Ca^2+^ concentrations ranging between 10^−9^ and 10^−8^ M, which are 1 to 2 orders of magnitude lower than the estimated steady-state [Ca2+]_cyt_. Furthermore, Ca^2+^ levels are high around the root tip, where auxin levels are high and CMI1 is expressed (Fig 1). Taken together, these data indicate that CMI1 should be constantly bound to Ca^2+^. Thus, rather than functioning as a Ca^2+^ sensor, CMI1 is likely a cell-specific transducer of the auxin response.

Studies carried out over the last several years have shown that rapid responses to auxin, which take place within minutes, depend on TIR1/AFB–Aux/IAA auxin receptors–coreceptors [1,26–28]. For these rapid auxin responses, the regulation of CMI1 expression by auxin and its biochemical properties, which allow it to function irrespective of the changes in Ca^2+^ levels, are intriguing. CMI1 is regulated by TIR1/AFB–Aux/IAAs and, upon its expression, can rapidly transduce nontranscriptional responses.

The phenotype of *cmi1* mutant plants is associated with impaired auxin responses (Figs 2 and 3). The interaction assays indicate that CMI1 interaction with ICR1 requires CBLD but that, at the same time, its interaction is highly specific, since CMI1 did not interact with other members of the ICR family, including ICR4, which is highly similar to ICR1. The binding specificity makes identification of CMI1-binding proteins by prediction extremely difficult, if not impossible. We have recently identified additional CMI1 interacting protein in high-throughput screens. However, the analysis of these proteins is beyond the scope of this work.

### The function of CMI1 and its interaction with ICR1

Ectopic expression of CMI1 resulted in root meristem collapse and inhibition of root growth (Fig 5). It appears therefore that the tight regulation of CMI1 expression is critical for plant development. Interestingly, coexpression of CMI1 with ICR1 suppressed the root growth inhibition phenotype (Fig 5), suggesting that ICR1 may function in restricting CMI1 signaling. ICR1 can affect the subcellular localization of CMI1 (Figs 5 and 9). In the root meristem, CMI1 levels are highest in the QC, where ICR1 is posttranslationally degraded [39,40], suggesting that CMI1 function in QC is different than in surrounding tissues. Auxin has complex roles in regulation of the interaction between CMI1 and ICR1 and, in turn, CMI1 function because it induces both the expression of CMI1 and the degradation of ICR1 [39,40]. Auxin or other factors may also regulate the cell-specific levels of other CMI1-interacting protein(s).

Although KIC and CMI1 do not share common binding partners, it is interesting that they both interact with MT-binding proteins (this work and [41]). Unlike KCBP, which is a kinesin with enzymatic activity, ICR1 is a coiled-coil domain protein that does not contain additional known catalytic or structural domains and likely functions as a scaffold [38]. KIC inhibits interaction of KCBP with MTs and its ATPase activity [41,67]. Data in this work indicate that ICR1 can recruit CMI1 to MTs, yet it is unknown whether CMI1 affects ICR1 interaction with MTs. Unfortunately, in vitro assays to test the effect of CMI1 on ICR1 MT binding were unsuccessful because of the requirement to include Ca^2+^ in the reaction medium, which in vitro leads to MTs destabilization.

### Structure–function of CMI1

Despite having a single Ca^2+^-binding EF-hand, CMI1 most likely interacts with ICR1 and possibly with other target proteins as a monomer. The 3D structure of CMI1 homologue KIC revealed existence of two EF-hands: a canonical Ca^2+^-binding EF-hand and a second EF-hand that lacks essential residues for divalent ion binding. Upon Ca^2+^ binding, both the Ca^2+^-binding and Ca^2+^ independent EF-hands form an open conformation, creating the hydrophobic pocket that can accommodate the KCBP CBD [67]. Our structural modeling, structure–function assays, and the SEC-MALS results indicate that like KIC, CMI1 has a canonical Ca^2+^-binding EF-hand and a Ca^2+^-independent EF-hand that enable it to function as a monomer. Possibly, the oligomerization of ICR1, which contains a long coiled-coil domain [38], may induce accumulation of CMI1 molecules at discrete cellular domains.

The identification of the hydrophobic pocket in CMI1 and its interaction with ICR1 via a CBLD suggest that CMI1 may interact with other proteins that contain a CBLD. Given that the interaction of CMI1 with the C-terminal ICR1 CBLD is weaker than its interaction with longer fragments of ICR1, it is likely that the binding specificity between the proteins is determined by additional residues in ICR1. Thus, it is difficult to predict which CBLD-containing proteins would interact with CMI1.

### Concluding remarks

The roles of Ca^2+^ as a second messenger in responses to diverse environmental stimuli have been extensively studied and discussed. Yet, the steady-state distribution of Ca^2+^ in the root and its close association with auxin distribution (this work and [24–26]) suggest that Ca^2+^ also has important functions in regulation of root development. This work identified CMI1 as a cell-specific transducer of the auxin-Ca^2+^ developmental signaling in the root.

## Materials and methods

### Molecular cloning

The plasmids used in this study are listed in supplemental information S1 Table. *pICR1>>GFP-ICR1* and *pCMI2>>GFP-ICR1* plants were previously described [39,40]. *pCMI1*::*CMI1-GUS* (*pSY1804*) was constructed by amplifying a 2,526-bp fragment containing the 2,040-bp promoter, 78-bp 5′-UTR, and the 408-bp CMI1 ORF, in which the TGA stop codon was changed to TAA (Leu). The resulting fragment was digested with *EcoRI* and *SalI* and cloned into *pENTRY1a*. The resulting plasmid *pSY1802* was recombined with *pMDC162* using LR clonase to obtain *pSY1804*. *pCambia2300-RFP-CMI1* (pSY1351) was generated by cloning mRFP upstream to the CMI1 ORF into *pCambia2300*. Transactivation CMI1 promoter plasmid (*pSY1806*) was constructed as follows: a 2,040-bp fragment of the *CMI1* promoter was amplified, digested with *Sal1*, and subcloned into *pLhG4Bj36* upstream of the chimeric transcription factor LhG4 to create plasmid *pSY1805*. *pSY1805* was then digested with *NotI*, and the resulting fragment containing *pCMI1*::*LhG4-terminator* was subcloned into *pART27* plant binary plasmid to obtain *pSY1806*. To obtain the *mRFP-CMI1 Op* reporter plasmid, the *mRFP-CMI1* fragment from *pSY1351* was digested with *HindIII* and *XhoI* and subcloned into *pOp* to obtain *pSY1807*. Subsequently, *pSY1807* was digested with *NotI*, and the resulting fragment containing *10XOp*::*mRFP-CMI1* was subcloned into the plant binary vector *pMLBART* to obtain *pSY1808*. pGAD-CMI1 was created as follows: the coding sequence of CMI1 was amplified from cDNA and subcloned into pGET (Thermo Fisher Scientific). It was then digested with *BamHI* and *SalI*, and the resulting fragment was ligated to pGAD vector to obtain *pSY1565*.

The generation of plasmids for yeast 2-hybrid and plant colocalization assays of site-directed and deletion mutants of CMI1 and ICR1 and plasmids for expression of CMI1 in *Escherichia coli* were carried out as follows. For site-directed mutagenesis (SDM), primers were designed using the QuikChange Primer Design tool found at Agilent website (https://www.genomics.agilent.com/primerDesignProgram.jsp). SDM was perform with *Pfu-Ultra* DNA polymerase (S2 Table) followed by digestion with *DpnI* (S2 Table) to eliminate unwanted templates. In two cases, when the SDM did not provide the desired mutants (cmi1^L92A^), an alternative approach of a three-step overlap extension PCR reaction using Phusion DNA polymerase (S2 Table) was performed. From this point, the cloning steps were the same as described below.

Genes of interest were cloned with flanking ends of attB1/2 recombination sites using a two-step reaction of Phusion high-fidelity DNA polymerase (S2 Table). In cases when several DNA fragments were observed in the PCR reaction products, the relevant band was extracted using QIAEX II gel extraction kit (QIAGEN) or Wizard SV Gel and PCR Clean-Up System (Promega) (S2 Table). The attB1/2 flanking genes were transferred into *pDONR221* using the BP clonase reaction (S2 Table). All clones were verified by sequencing.

For yeast 2-hybrid, constructs were transferred by recombination from *pEntry221* and then by recombination to *pDEST22* (prey) or *pDEST32* (bait) using the LR clonase (S2 Table). Bait and prey plasmids were transformed into *PJ69-4a* yeast strain. Presence of respective plasmids was verified by yeast colony PCR (S2 Table).

For colocalization assays in plants, *CMI1*, *cmi1*^*L59A*^, and *cmi1*^*D85N*^ were transferred by recombination from *pEntry221* to *pGWB6-35S*::*eGFP* using the LR clonase (S2 Table). In addition, 3-way GATEWAY recombination reactions (S2 Table) were carried out with *pEntryP4-P1R-35S* promoter, *pEntry221-ICR1* or *pEntry221-icr1*^*W338A*^ (both without stop codon), and *pEntryP2R-P3-mCherry* into pB7m34GW. Plasmids were verified by colony PCR (S2 Table) and sequencing. For expression in plants, plasmids were transformed into *Agrobacterium tumefaciens* stain *GV3101 pMP90*.

#### Cloning for protein expression in *E*. *coli*

A PCR product of CMI1 with flanking BamH1 and Not1 sites was subcloned into pJET1.2 using the CloneJET PCR cloning kit (S2 Table). The resulting plasmid was digested with BamH1 and Not1, and the CMI1 fragment was subcloned into *pET21d*.*H8*.*Nia*.*yBRFc*.*T*.*GSTrc* digested also with BamH1 and Not1 to isolate the *pET21d-His*_*8*_*-TEV* fragment. The resulting plasmid *pSY2408* (*pET21d_His8-TEV-CMI1*) was designed to express His_8_-TEV-CMI1 fusion protein that enables purification of CMI1 on a metal chelate Ni-column and cleavage of the His_8_-tag by TEV protease.

### Plant material and growth conditions

The *Arabidopsis* transgenic lines used in this study are listed in supplemental information S3 Table. Long-day grown (16 hours light/8 hours dark, 22°C) *Arabidopsis Columbia-0* (*Col-0*) and *Ler* ecotypes were used for stable expression, mutant phenotypic analysis, protein localization, and Ca^2+^ measurement. *Arabidopsis*, *mRFP-CMI1*, and *pCMI1>>mRFP-CMI1* and *pICR1>>mRFP-CMI1* plants were generated by crossing *pCMI1-LhG4* to *pop-mRFP-CMI1* and *pICR1-LhG4* to *pop-mRFP-CMI1*. The *cmi1* mutant (CSHL_GT24505) is in the *Ler* background. To analyze *DR5*::*GFP*_*rev*_ and *pICR1>>GFP-ICR1* expression in the *cmi1* mutant background, *DR5*::*GFP*_*rev*_ and *pICR1>>GFP-ICR1* were crossed into WT *Ler* and *cmi1* backgrounds. M3 generation WT or *cmi1* homozygote mutant plants that harbored the *erecta* phenotype and expressed either *DR5*::*GFP*_*rev*_ or *pICR1>>GFP-ICR1* were selected for further analysis. Quantification of fluorescent signals was performed using Image J. For *DR5*::*GFP*_*rev*_ quantification, we used 12–16 images of independent root tips when 2–4 QC cells are in the center. Cell layers 1–6 were defined from QC to the last columella layer, and GFP signal intensity was measured in the same area (below the QC cells) in each layer using Image J. The average of the GFP intensity is presented in the graph, and the bars are the standard error (SE) (Fig 6). To quantify the ectopic expression of GFP-ICR1 in the QC cells of *cmi1* mutant, 18–20 roots of each WT (*Ler*) or *cmi1* plants were imaged when QC cells (2–4 cells) are visible in the center. The number of QC cells in which a GFP-ICR1 signal was detected was used to calculate the percentage of the roots with or without ectopic expression. Complementation of *cmi1* was performed by crosses with *pCMI1*::*CMI1-GUS* plants. The analysis was performed using nonsegregating lines from the fourth and fifth generations. For Ca^2+^ imaging, the *pUBQ10*::*YC3*.*6* Yellow Cameleon [35] was transformed into *Ler* WT and *cmi1* plants. Several independent transgenic lines were used for the Ca^2+^ imaging.

### Protein expression and antibody generation

Expression in *E*. *coli* Rosetta (DE3) and purification of recombinant His_6_-CMI1, His_6_-ICR1, and GST-ICR1 were carried out according to standard protocols using Ni-NTA (Qiagen) and Glutathione sepharose (GE) resins, as previously described [38,69]. His_8_-TEV-CMI1 was purified over Ni-NTA (Quiagen). Eluted fractions were passed through HiPrep 26/10 desalting column (GE Healthcare) with the extraction buffer (50 mM sodium phosphate buffer [pH 7.5], 300 mM NaCl, and 1 mM DTT) to ensure flushing of imidazole presence from the elution buffer (50 mM sodium phosphate buffer [pH 7.5], 300 mM NaCl, 1 mM DTT, and 250 mM imidazole). Eluted fractions were incubated overnight with His_6_-tagged TEV protease at 4°C followed by purification over a second Ni-NTA. The untagged CMI1 was collected from the flow-through and concentrated with Amicon Ultra-15 with molecular weight cutoff (MWCO) of 3 kDa (Millipore) at 4,000*g* and 4°C to a final volume of approximately 500 μl. The concentrated protein samples were filtrated through Millex 0.22-μm syringe filter (MILLIPORE) and uploaded onto a gel filtration column of HiLoad 16/600 Superdex 200 pg (GE Healthcare) and eluted with a gel filtration column buffer (60 mM MOPS [pH 7.2], 200 mM KCl, and 2 mM DTT). Purified proteins were concentrated using Amicon Ultra-15 with MWCO of 3 kDa at 4,000*g* and 4°C, divided into aliquots, batch frozen in liquid nitrogen, and kept at −80°C until further use.

Anti-CMI1 antibodies were raised in rabbits. Ni-NTA-purified His_6_-CMI was further purified by SDS-PAGE. The His_6_-CMI1 band was eluted from the gels and were used for rabbit immunization.

### In vitro ICR1-CMI1 and ICR1-ICR1 interaction assays

Pull-down of His_6_-CMI1 or HiS_6_-ICR1 with GST-ICR1: 1.2 μg GST-ICR1 or 0.4 μg GST was mixed with 100 μL of phosphate buffer saline (PBS), 1% Triton X-100, and 10 μL of Glutathione sepharose slurry and incubated with shaking for 30 minutes at room temperature (RT). The beads were then washed 3X with PBS, 1% Triton X-100, and were adjusted in Ca^2+^/EGTA reaction buffer: 20 mM Tris-HCl (pH 7.5), 5 mM CaCl_2_/10 mM EGTA, 0.1 mg/mL BSA, 200 mM NaCl, 1% Triton X-100. His_6_-CMI1 (0.5 μg) was added for pull-down of His_6_-CMI1 by GST-ICR1. Alternatively, 0.09/0.45/1 μg His_6_-CMI1 and 0.5 μg His_6_-ICR1 were added for pull-down of HiS_6_-ICR1 by GST-ICR1. The reaction volumes were then adjusted to 100 μL with the respective buffer. The mixtures were incubated with shaking for 1 hour at RT. Subsequently, the beads were precipitated and washed 1X with wash buffer 1–20 mM Tris-HCl (pH 7.5), 5 mM CaCl_2_/10mM EGTA, 0.1 mg/mL BSA, 1M NaCl, 1% Triton X-100—and 4X in wash buffer 2–20 mM Tris-HCl (pH 7.5), 5 mM CaCl_2_/10mM EGTA, 0.1 mg/mL BSA, 200 mM NaCl, 1% Triton X-100. The beads were then precipitated and resuspended in SDS-PAGE sample buffer, and the proteins were resolved by SDS-PAGE [70].

For coimmunoprecipitation of His_6_-ICR1 and His_6_-CMI1 with anti-CMI1 antibodies, His_6_-CMI1 and His_6_-ICR1, 1 μg of each, were incubated with shaking in 300 μL of Ca^2+^/EGTA reaction buffer for 1 hour at RT. Subsequently, 1 μL of anti-CMI1 antibodies was added, and the mixture was further incubated with shaking for 2 hours at RT. A Protein A bead (10 μL; Adar Biotech #1016–5) slurry in Ca^2+^/EGTA reaction buffer was added, and the mixture was further incubated with shaking for 1 hour at RT. Subsequently, the beads were washed 3X with 1 mL ice-cold Ca^2+^/EGTA reaction buffer and resuspended in SDS-PAGE sample buffer, and proteins were resolved by SDS-PAGE. Proteins were detected by immunoblots decorated with mouse anti-poly-His monoclonal antibodies (Sigma H-1029) and goat anti-mouse horse radish peroxidase (HRP)-conjugated secondary antibodies (BioRad).

### CD-spec

Protein samples were dialyzed overnight in buffers contained 10 mM Tris-H_2_SO_4_ (pH 7.5), 25 mM KCl, and 200 μM DTT. Buffers also contained CaCl_2_ and EDTA at different concentrations to obtain the desired Ca^2+^-free concentrations (Table 1). All protein samples and buffers were filtrated before use through a Millex 0.22-μm syringe filter (MILLIPORE) or Stericup 0.22-μm vacuum filtration system (MILLIPORE), respectively. Protein concentration was determined using a Bradford assay standard curve for BSA. Cuvette path length was 0.1 mm, and samples’ concentrations were 60 μM. Measurements were performed using a Chirascan CD spectrometer (Applied Photophysics), ranging between 180 nm and 260 nm at 21°C. Using the Pro-Data Viewer software (https://www.photophysics.com), each spectrum was averaged from five repeated scans. Then, raw data were corrected by subtracting the contribution of the buffer to the signal, and subtracted data were smoothed (5-nm window) and exported to Excel. In Excel, data converted from observed ellipticity to mean residue ellipticity (MRE) units using the following equation:

$$MRE\;(deg*{cm}^{2}*{dmol}^{-1})=\frac{observed\;ellipticity\;(millide\;gree)}{pathlength\;(mm)\times concentration\;of\;protein\;(M)\times number\;of\;residues}$$

**Table 1:** CaCl_2_ and EDTA composition in the CD spectroscopy buffers.

Buffer name	2 mM CaCl_2_	*200 μM Ca^2+^ free	*2 μM Ca^2+^ free	*200 nM Ca^2+^ free	*20 nM Ca^2+^ free	*2 nM Ca^2+^ free	1 mM EDTA
CaCl_2_	2 mM	1,199.9 μM	998.8 μM	969.5 μM	759.7 μM	240.2 μM	-
EDTA	-	1 mM	1 mM	1 mM	1 mM	1 mM	1 mM

*Calculations were made using the WEBMAXC EXTENDED server (http://web.stanford.edu/~cpatton/maxc.html).

Abbreviation: CD, circular dichroism.

All measurements were repeated at least thrice. The α-helical content of sampled proteins was extracted from MRE values at 222 nm using the following equation [71]:

$$\alpha -helix\;(\%)=\frac{{\left[\theta \right]}_{222}(deg*{cm}^{2}*{dmol}^{-1})}{-40,000\times (1-\frac{4.6}{number\;of\;residues}}\times 100$$

The α-helical content was averaged from the three repetitions, and SE was calculated as well.

### SEC-MALS

The SEC-MALS buffer containing 10 mM Tris-HCl (pH 7.5), 25 mM KCl, 200 μM DTT, and 2 mM CaCl_2_ was double-filtrated through Stericup 0.22-μm vacuum filtration system (Millpore) and then through Whatman Anodisc 0.02-μm Filter Membranes (GE Healthcare). Protein samples were filtrated through Whatman Anotop 10 Plus 0.02-μm syringe filter (GE Healthcare), and their concentration was determined using a Bradford assay standard curve for BSA. Protein samples were injected into a Shodex KW404-4F column (Shodex) equilibrated overnight with the buffer above. The Agilent 1200 Series HPLC System (Agilent Technologies) was coupled with a DAWN HELEOS II light scattering detector (Wyatt Technology) and an Optilab rEX refractive index detector (Wyatt Technology). Molecular mass analyses were performed using the ASTRA software (https://www.wyatt.com/products/software/astra.html). Data were exported from the ASTRA software in order to build the graphs in Excel.

### Homology modeling of CMI1

Amino acids sequences of KIC and CMI1 were underwent a multiple sequence alignment (MSA) using the MUSCLE algorithm (https://www.ebi.ac.uk/Tools/msa/muscle). The MSA results were converted into PIR format with the necessary adjustments to the solved crystal structure of KIC-KCBP complex (PDB ID code 3H4S) [67]. A pairwise alignment of KIC with CMI1 was extracted from the MSA PIR format and run using the Modeller 9.19 (https://toolkit.tuebingen.mpg.de/#/tools/modeller). The built model of CMI1 was examined using the WHAT_CHECK (SAVES 5.0 server [http://servicesn.mbi.ucla.edu/SAVES]). The CMI1 model was visualized and edited using PyMOL (https://www.schrodinger.com/pymol) and Adobe Photoshop CS6.

### Yeast two-hybrid assays

*Saccharomyces cerevisiae* strains Y190 and *PJ69-4a* were used as hosts. *pGAD-CMI1* or *pGAD-cmi1D85N* plasmids were cotransformed with *pGBT-ICR1*/*pGBT-ICR2*/*pGBT-ICR4* into yeast cells via a standard lithium acetate transformation protocol. Colonies expressing both plasmids were grown on a medium lacking leucine (Leu), tryptophan (Trp), and histidine (His) and containing 50 mM 3-Amino-1,2,4-triazole (3-AT). In addition, β-galactosidase activity assays were performed. Each test was carried out with at least four independent transformants. Assays of site-directed mutants in CMI1 and ICR1 and deletion mutants of ICR1 were carried out with *PJ69-4a* yeast. The optical density in 600 nm (OD_600_) was measured and diluted into OD_600_ of 0.5. From this yeast suspension (referred to as 1), four decimal dilutions were made (1:10, 1:100, 1:10^3^, and 1:10^4^). From each dilution, a drop of 5 μl was placed on -LT (S2 Table) and -LTH (S3 Table) with YNBx1, 2% glucose, and 1 mM 3-AT plate. The plates were incubated at 21°C.

### Plant protein colocalization assays

Colocalization assays were performed using transient expression of tested proteins by transforming *A*. *tumefaciens GV3101 pMP90* cells harboring the respective plasmids into the abaxial side of *N*. *benthamiana* leaf epidermis essentially as previously described [38,72] with the following modifications. In cases when expression levels were too low for detection, *Agrobacterium* expressing the silencing suppressor protein p19 form tomato bushy stunt virus [73] were cotransformed, added at a dilution OD_600_ of 0.05. Following transformation, plants were maintained in a growth room for 48 hours prior to imaging.

### Immunostaining

Immunostaining of PIN1 and PIN2 in *Col-0* WT *and cmi1* mutant roots was carried out essentially as previously described [74]. Primary antibodies used in this study were anti-PIN1 (1:1,000; sc-27163; Santa Cruz Biotechnology) and anti-PIN2 (1:400; N782248; NASC). Anti-rabbit Cy3 (1:600; CALTAG Laboratories, Invitrogen) and AlexaFluor 488 anti-rat (1:600; Invitrogen) were used as secondary antibodies. Fluorescence was observed using a Zeiss LSM780-NLO confocal microscope/multiphoton microscope. Cy3 was observed by excitation at 543 nm and emission at 560 nm and AlexaFluor by excitation at 488 nm and emission at 499–519 nm. Quantification of PIN2 relocation was performed by scoring the number of cells with different PIN polarities.

### RNA extraction and qPCR

For auxin induction experiments, seedlings were grown vertically on 0.5X Murashige Skoog (MS) supplemented with 1% sucrose for 5 days. Before treatment, seedlings were transferred to liquid 0.5X MS with 1% sucrose for 2 hours in a growth chamber. The 0.5X MS medium was then replaced with fresh 0.5X MS medium (mock) or 0.5X MS medium containing 1 μM of NAA. Following 2 hours of incubation, seedlings were frozen in liquid nitrogen, and total RNA was extracted using the RNAeasy kit (Qiagen). PP2A was used as a reference gene. The qPCR data were normalized to the reference gene. Three biological replicates with four technical replicates were carried out for each treatment. The qPCR program was as follows: 10 minutes at 95°C, followed by 40 cycles of 15 seconds of denaturation at 95°C, 1 minute annealing, and elongation at 60°C. The results were analyzed using the StepOne software.

### Confocal imaging

Confocal imaging was performed using Zeis780-NLO confocal laser scanning microscope (Zeiss, Jena, Germany) with 40X air and 20X, 40X, and 63X water immersion objectives with NAs of 0.75, 0.8, 1.0, and 1.2, respectively. Proteins tagged with eGFP or GFP were visualized by excitation with an argon laser at 488 nm. Emission was detected with a spectral GaAsP detector set between 499 nm and 552 nm. Proteins tagged to mCherry or mRFP were visualized by excitation with an argon laser at 561 nm and spectral GaAsP detector set between 579 nm and 632 nm. Image analysis was carried out with Zeiss ZEN 2012 (https://www.zeiss.com/microscopy/int/software-cameras.html) and Adobe Photoshop CS6 (https://www.adobe.com), Fiji (Image J) (https://fiji.sc/), and Imaris 8.4.1 (Bitplane).

### Microarray experiments

*Arabidopsis* seedlings were grown hydroponically for 6 days and subjected to auxin treatments as previously described [75]. Roots were collected after 30 minutes of exposure to either 20 nM IAA (“low auxin”), 20 μM IAA (“high auxin”), or conditioned media (“mock”). Affymetrix ATH1 arrays were hybridized with probes generated from total RNA of four biologically independent samples per treatment. Data shown are mean signal values with standard deviation.

### *Arabidopsis* sample preparation and Ca^2+^ imaging

Experiments were carried out essentially as previously described [35]. Surface-sterilized *Arabidopsis Ler* WT or *cmi1* (3 independent lines for each) seeds expressing UBQ10-YC3.6 were plated on 0.5X-strength MS medium (Duchefa) containing 1% (w/v) sucrose, solidified with 0.8% agar (Duchefa) (pH 5.8), and stratified for 2 days in the dark at 4°C. The plates were transferred to a growth chamber (16 hours 22°C: 8 hours 18°C, light: dark; 120–150 μmol m^−2^ s^−1^ light intensity), and seeds were grown vertically for 5–7 days. Single *Arabidopsis* seedlings (5–7 days old) were placed inside a custom-made flow-through chamber (or perfusion chamber) containing imaging buffer (5 mM KCl, 10 mM MES, and 10 mM CaCl_2_ (pH 5.8), adjusted with Tris). The seedling was fixed inside the chamber with cotton wool soaked in the imaging buffer as previously described [35,76]. The chamber was placed on the stage of an inverted ZEISS Axio observer (Carl Zeiss Microimaging GmbH, Goettingen, Germany) equipped with an emission filter wheel (LUDL Electronic Products, Hawthorne, NY, USA) and a Photometrics cool SNAPHQ2 CCD camera (Photometrics, Tucson, AZ, USA). A Zeiss Plan-APOCHROMAT 20/0.8 dry objective of the microscope was used for imaging. A xenon short-arc reflector lamp (Hamamatsu) with a 440-nm filter provided the excitation. Emission filters used were 485 nm (CFP) and 535 nm (YFP). A peristaltic pump was used for buffer circulation inside the flow-through chamber with a flow rate of 1.5 ml min^−1^. YFP and CFP images were taken at 6-s intervals using the METAFLUOR software (Meta Imaging series 7.7; Molecular Devices, Downingtown, PA, USA). After monitoring the root in the buffer (continuous flow-through) for 2 minutes, the buffer was replaced by a buffer containing 10 μM NAA (Sigma Aldrich) for 7 minutes.

### Ca^2+^ imaging data analysis

Offline calculation of the FRET ratio was performed using ImageJ64 software (http://rsb.info.nih.gov/ij/) with the RATIOPLUS plug-in. The intensities of CFP and YFP were measured from single CFP and YFP images as pixel intensity in arbitrary units. The ratio between YFP emission and CFP emission was calculated after background subtraction. We calculated the change in ratio R_t_-R_0_ or ΔR, where R_0_ is the basal ratio before application of the stimulus and R_t_ is the ratio at a specific time point. We normalized the ΔR to the basal ratio value (ΔR:R_0_) and plotted ratio graphs for each measurement. We aligned all the graphs to their first response point and plotted averaged ratio graphs.

### Analysis of Ca^2+^ responses

The Ca^2+^ peaks were divided into low, middle, and high threshold peaks, depending on the ratio change presented as height of the peak from the base. Peaks with a ratio change of 0 to 0.1 were considered as low-threshold peaks, a ratio change of 0.1 to 0.15 as intermediate, and the peaks with ratio changes higher than 0.15 were considered as high threshold peaks. Percentage was then calculated. Fifteen to 17 seedlings were analyzed for each genotype. The average ratio graphs were calculated from six to seven measurements.

### High-resolution Ca^2+^ imaging

High-resolution imaging was performed as previously described [76], with a Leica DMI 6000B inverted microscope equipped with a Leica TCS SP5 laser scanning device and HDy, using the Leica confocal software (Leica Application Suite–Advanced Fluorescence 2.6.0.7266; Leica Microsystems, Wetzlar, Germany). For excitation, an argon laser with a 458-nm line was used. The CFP and FRET emissions were collected at 473–505 and 526–536 nm, respectively. Images were acquired with a 25x objective (HCW RAPO L 25.0 × 0.95 water). Image acquisition was conducted as follows: scanning speed (400 Hz), image dimension (512 × 512), pinhole (2–4 airy unit), and line average (4). YFP and CFP images were acquired as a time series in a 6-s interval. Offline calculation of the FRET ratio was performed using ImageJ RATIOPLUS plug-in.

### Data analysis and statistics

The expression pattern of pCMI1::CMI1-GUS was tested in 12 transgenic lines; we present here line 1, which has a WT phenotype and segregates 1:3. The induction of CMI1 expression with auxin was reproduced more than 10 times in the lab by different people.

The microarray experiments were done with four biologically independent samples per treatment.

Ca^2+^ imaging was carried out with three independent lines both of WT and *cmi1* background; 15–17 seedlings were analyzed for each genotype. The average ratio graphs were calculated from six to seven measurements. Error bars represent SE.

The root length was measured in 38 and 41 seedlings for the WT and *cmi1*, respectively. For the root meristem measurements, 18 and 15 images of the roots with propidium iodide (PI) staining were used.

Complementation assays of *cmi1* with pCMI1::CMI1-GUS were analyzed on 30–40 seedlings from each phenotype. This root assay was done 6 times with similar results.

Hypocotyl length was measured using image J in 30–50 seedlings of each genotype. This experiment was repeated more than five times with similar results.

The measurements of root hairs were performed using Image J on 20 seedlings from each genotype sampling 20–30 root hairs from each seedling.

Auxin response marker intensity was measured using Image J in different cell layers of 10–12 images of root tips expressing *DR5*::GFP. Identical confocal settings were used for the imaging of all samples, and the fluorescence intensity was measured for identical square areas so that it could be compared and normalized.

GFP-ICR1 abundance in the QC cells was examined in 14–20 root tips of WT or *cmi1* expressing *pICR1*>>GFPICR1. For quantification, QC cells expressing GFP-ICR1 were counted.

Immunostaining with anti-PIN1 and PIN2 antibodies was reproduced three times each with 10–15 roots for each genotype.

Protein–protein interactions in yeasts were tested 4 to 10 times (each time with four different colonies for the assay).

In vitro assays were done 3 times with similar results.

Negative results with cmiD85N-ICR1 were reproduced in three independent experiments with four replicates each time.

All experiments in *N*. *benthamiana* were repeated at least three times; the representative images were used for preparation of the figures.

The means and the SEs were calculated using Excel. For statistical analysis when two groups were compared, we used the *t* test to calculate the *p*-value (using IBM SPSS 23 Statistics). *p*-Values of the relevant tests are noted in the figure legends.

## Supporting information

S1 Fig Auxin induces specific Ca2+ pattern. Epifluorescent images of root expressing the YC3.6-free Ca^2+^ sensor prior to auxin treatment (mock) and shortly after treatment with 10 μM NAA (NAA). NAA, 1-Naphthaleneacetic acid; YC3.6, Yellow Cameleon 3.6.
(TIF)

S2 Fig Expression of CMI1 is induced by auxin. qPCR showing induction of CMI1 expression 6 hours after treatment with mock or 10 μM IAA. CMI1, Ca^2+^-dependent modulator of ICR1; IAA, indole-3-acetic acid; qPCR, quantitative PCR.
(TIF)

S3 Fig CMI1-GUS can complement root growth inhibition in *cmi1*-knockout plants. (A) Primary root length of 7-day-old seedlings. Error bars are SE. (B) Representative hypocotyls and (C) root hairs used for quantifications presented in Fig 5L and 5K, respectively. CMI1, Ca^2+^-dependent modulator of ICR1; GUS, β-glucuronidase.
(TIF)

S4 Fig Lateral root development in wild type and *cmi1*. (A) Wild-type LRIs at different developmental stages. (B) *cmi1* LRIs. Note the abnormal LRI patterning. The developmental stages of the LRIs are noted. *cmi1*, Ca^2+^-dependent modulator of ICR1; LRI, lateral root initial.
(TIF)

S5 Fig PIN2 auxin efflux transporter is altered in *cmi1*. (A) Immunolocalization of PIN1 in the endodermis (“en”) and PIN2 in the cortex (“co”) and the epidermis (“ep”) in *Col-0* (WT) and *cmi1*. Arrowheads highlight the basal localization of PIN2 in WT cortex and apical and apolar localization in *cmi1* cortex. (B) Quantitative analysis of PIN2 distribution. Scale bar 20 μm. Error bars SE. Underlying data for this figure can be found in S2 Data. *cmi1*, Ca^2+^-dependent modulator of ICR1; *Col-0*, *Columbia-0*; PIN, PINFORMED; WT, wild type.
(TIF)

S6 Fig CMI1 is localized in the plasma membrane (“M”), cytoplasm (“C”), and nuclei (“N”) and sensitivity of its expression to salt stress. (A) Subcellular localization of mRFP-CMI1 in *Arabidopsis* cotyledon pavement cells. Localization of mRFP-CMI1 in the plasma membrane can be seen following plasmolysis (right panel). (B) Protein immunoblot decorated with anti-CMI1 antibodies showing the distribution of CMI1 between the soluble and insoluble fraction in the specified tissue samples. (C) The sensitivity of the anti-CMI1 antibodies as determined by protein immunoblot of the specified amounts of His_6_-CMI1. CMI1, Ca^2+^-dependent modulator of ICR1; mRFP, monomeric red fluorescent protein.
(TIF)

S7 Fig Amino acid sequence of CMI1, its induction by auxin, and its Ca2+-dependent pull-down by ICR1. (A) The amino acid sequence of CMI1. The loop region of the single EF-hand is underlined, and the D85 critical for Ca^2+^ binding is highlighted in red. (B) Protein immunoblot decorated with anti-poly-His antibodies showing that pull-down of His_6_-CMI1 by GST-ICR1 is specific and Ca^2+^-dependent. (C) Coomassie brilliant blue–stained SDS-polyacrylamide gel showing specified *E*. *coli* expressed and purified recombinant proteins used for the pull-down and immunoprecipitation assays (Fig 1). Numbers denote M_*r*_ in kDa. CMI1, Ca^2+^-dependent modulator of ICR1; ICR1, interactor of constitutively active ROP.
(TIF)

S8 Fig CMI1 exists as a monomer in solution. An SEC-MALS elution profile of 2 μg CMI1 in 2 mM Ca^2+^ solution. CMI1 eluted as a single peak with a molecular mass (red line) corresponding to a monomeric form. The profile is identical to that obtained with 4 μg protein (Fig 2E). Underlying data for this figure can be found in S2 Data. CMI1, Ca^2+^-dependent modulator of ICR1; SEC-MALS, size-exclusion chromatography multiangle light scattering.
(TIF)

S9 Fig CMI1 displays weak self-interaction in yeast two-hybrid assays. Yeast two-hybrid assays were carried out in the LexA system. In CMI1 self-interaction assays, weak X-Gal activity was evident after 48 hours. Strong X-Gal activity was observed in assays with ICR1 and no activity with the vector control. CMI1, Ca^2+^-dependent modulator of ICR1; ICR1, interactor of constitutively active ROP.
(TIF)

S10 Fig Structural modeling predicts that L-to-A mutations in CMI1 hydrophobic pocket do not induce structural changes. Structural models of CMI1 (blue) aligned with structural models of its hydrophobic pocket mutants; L59A (cyan), L92A (magenta), and L100A (yellow). The structure of main core of the CMI1 containing its hydrophobic pocket is not affected by the mutated residues (orange sticks). The structural deviations in the N- and C-terminal domains likely result from the low confidence of the model in these regions of the protein. CMI1, Ca^2+^-dependent modulator of ICR1.
(TIF)

S11 Fig Interaction between CMI1 and icr1W266Q is similar to icrW266A. Numbers above the panel denote dilution order. -LT, Leu- and Trp-dropout medium; -LTH: Leu-, Trp-, and His-dropout medium.
(TIF)

S12 Fig ICR1 is localized on MTs. (A-C) ICR1-mCherry (ICR1) is colocalized to MTs with TUA6-GFP MT marker on MTs. (D-F) Localization of ICR1 and GFP-CMI1 (CMI1) on MTs is sensitive to the anti-MT drug oryzalin. O/L mCherry/GFP overlay. Bar: 20 μm. CMI1, Ca^2+^-dependent modulator of ICR1; GFP, green fluorescent protein; ICR1, interactor of constitutively active ROP; MT, microtubule; TUA6, Tubulin alpha-6.
(TIF)

S1 Table Plasmids used in this study. (DOCX)

S2 Table Materials used in this work. (DOCX)

S3 Table *Arabidopsis thaliana* lines used in this study. (DOCX)

S1 Data Numerical data related to main text figures. (XLSX)

S2 Data Numerical data related to SI figures. (XLSX)

## Abbreviations

ABCB: ATP-binding cassette B
AD: activation domain
ARF: auxin response factor
*axr1*: auxin-resistant 1
CaM: calmodulin
CBD: CaM-binding domain
CBL: calcineurin B-like
CDPK: Ca^2+^-dependent kinase
CD-spec: circular dichroism spectroscopy
CMI1: Ca^2+^-dependent modulator of ICR1
CML: CaM-like
CNGC14: cyclic nucleotide gated channel 14
CSHL: Cold Spring Harbor Laboratory
FRET: fluorescence resonance energy transfer
Gal4-DB: Gal4 DNA-binding domain
GFP: green fluorescent protein
GST: glutathione S-transferase
GUS: β-glucuronidase
IAA: indole-3-acetic acid
ICR1: interactor of constitutively active ROP
KCBP: kinesin-like CaM-binding protein
KRP1: KIC-related protein 1
LAX: Like AUX1
*Ler*: Landsburg *erecta*
LR: lateral root
LRC: LR cap
mRFP: monomeric red fluorescent protein
MT: microtubule
NAA: 1-Naphthaleneacetic acid
NRT1.1: nitrate transporter 1.1
PBP1: PID-binding protein 1
PID: PINOID
PIN: PINFORMED
PLT: PLETHORA
QC: quiescent center
qPCR: quantitative PCR
ROP: Rho of plants
SCF^TIR1/AFB^: Skp Cullin F-box transport inhibitor response 1
SCN: stem cell niche
SEC-MALS: size-exclusion chromatography multiangle light scattering
TCH3: TOUCH3
TUA6: Tubulin alpha-6
WT: wild type
YC3.6: Yellow Cameleon 3.6
