# Supplementary figures and images for: A novel Ca2+-binding protein that can rapidly transduce auxin responses during root growth
Source: PLoS Biol. 2019 Jul 11;17(7):e3000085. doi: 10.1371/journal.pbio.3000085 (PMC6650080; doi:10.1371/journal.pbio.3000085)

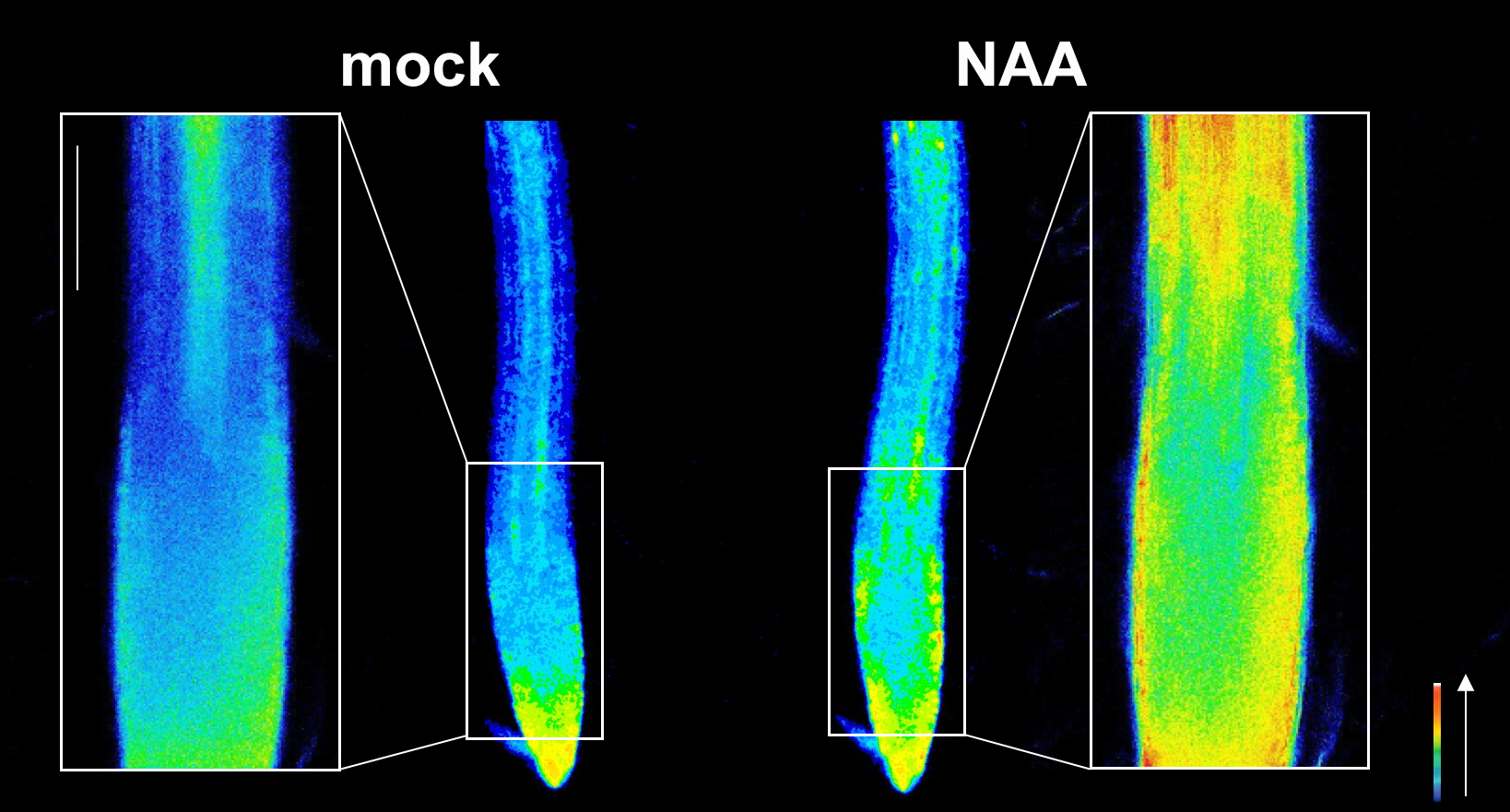

Supplement: S1 Fig — Epifluorescent images of root expressing the YC3.6-free Ca2+ sensor prior to auxin treatment (mock) and shortly after treatment with 10 μM NAA (NAA). NAA, 1-Naphthaleneacetic acid; YC3.6, Yellow Cameleon 3.6. (TIF) [file pbio.3000085.s001.tif]

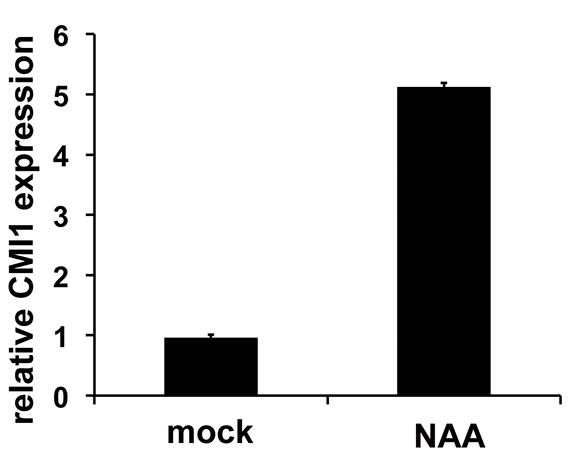

Supplement: S2 Fig — qPCR showing induction of CMI1 expression 6 hours after treatment with mock or 10 μM IAA. CMI1, Ca2+-dependent modulator of ICR1; IAA, indole-3-acetic acid; qPCR, quantitative PCR. (TIF) [file pbio.3000085.s002.tif]

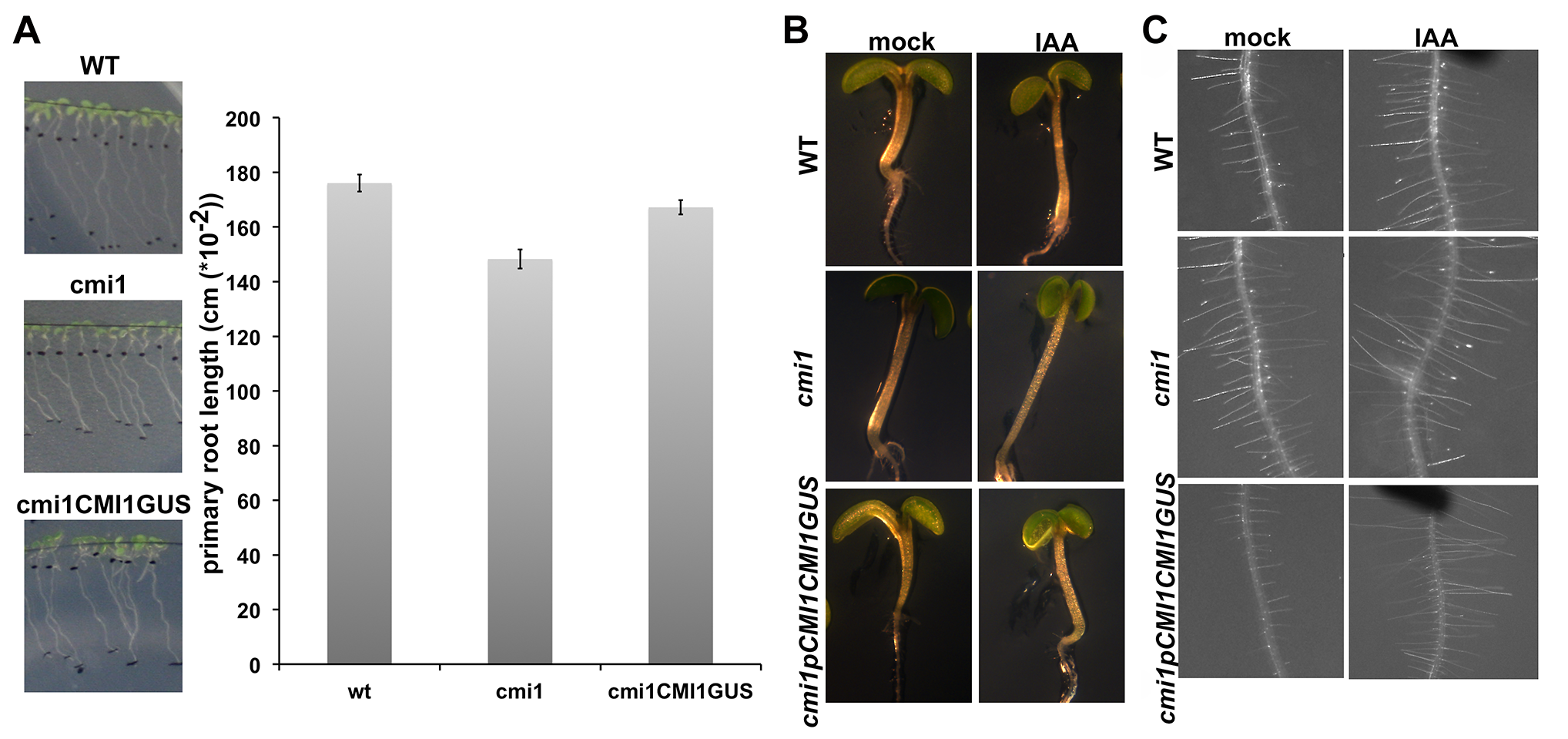

Supplement: S3 Fig — (A) Primary root length of 7-day-old seedlings. Error bars are SE. (B) Representative hypocotyls and (C) root hairs used for quantifications presented in Fig 5L and 5K, respectively. CMI1, Ca2+-dependent modulator of ICR1; GUS, β-glucuronidase. (TIF) [file pbio.3000085.s003.tif]

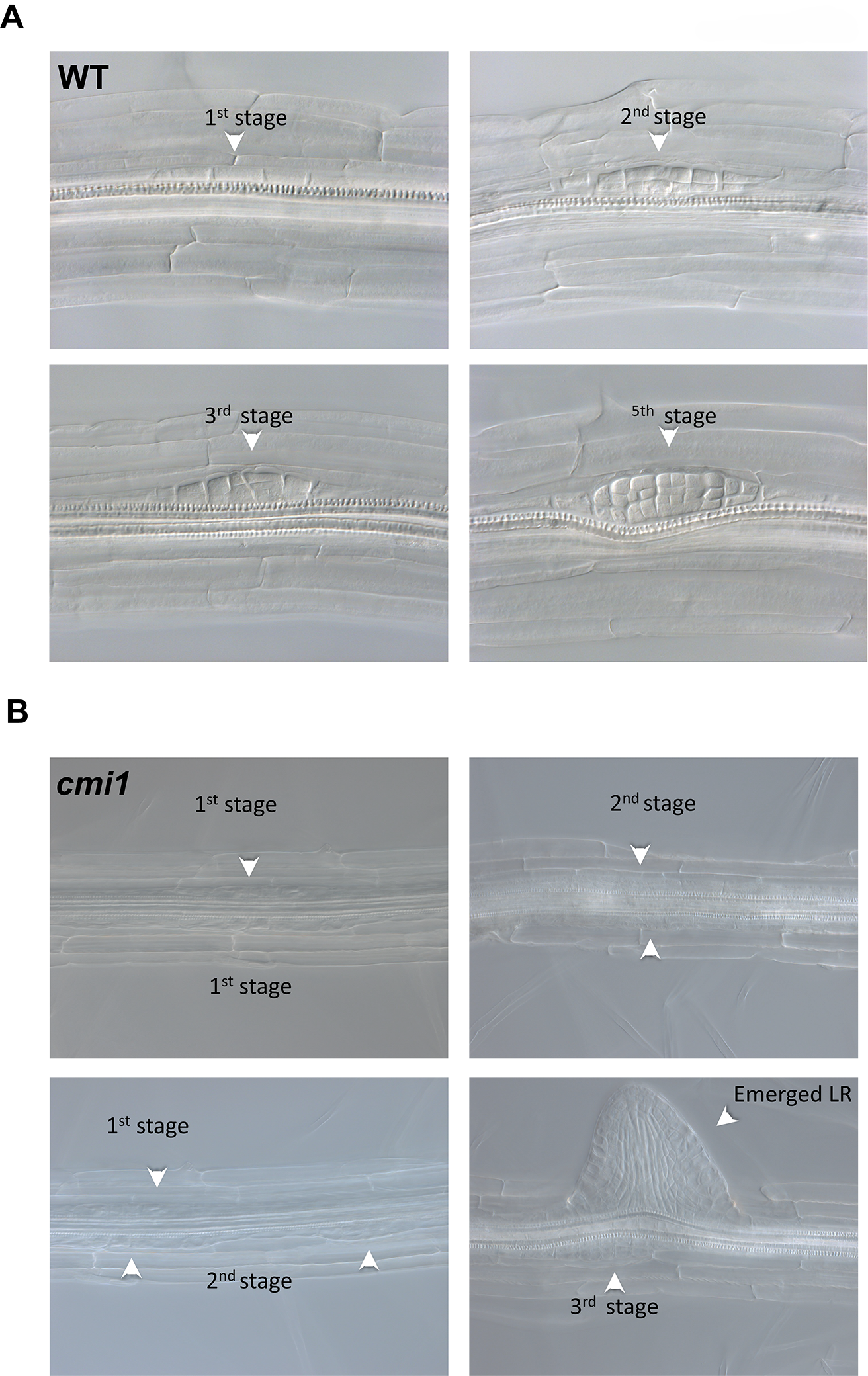

Supplement: S4 Fig — (A) Wild-type LRIs at different developmental stages. (B) cmi1 LRIs. Note the abnormal LRI patterning. The developmental stages of the LRIs are noted. cmi1, Ca2+-dependent modulator of ICR1; LRI, lateral root initial. (TIF) [file pbio.3000085.s004.tif]

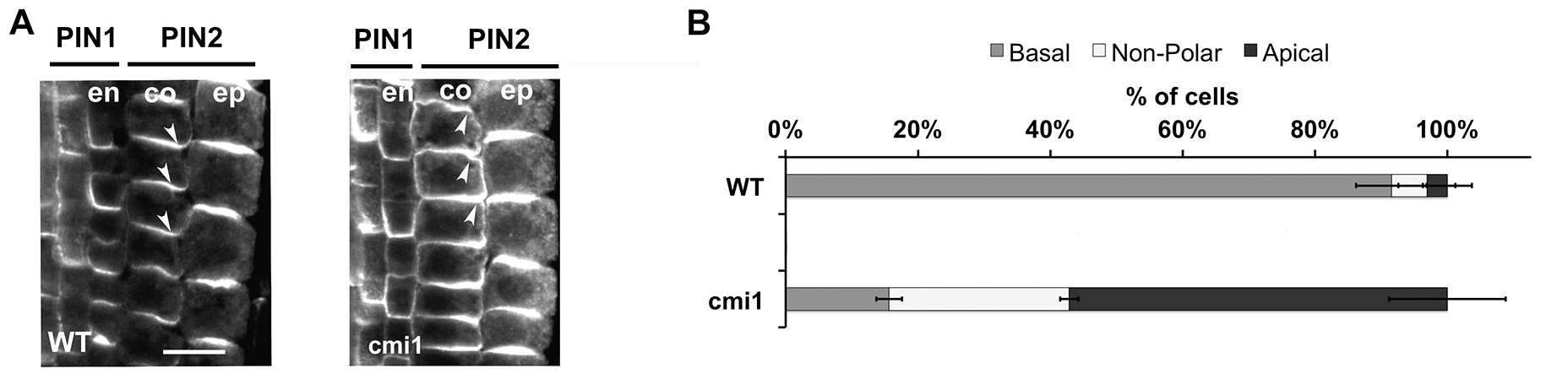

Supplement: S5 Fig — (A) Immunolocalization of PIN1 in the endodermis (“en”) and PIN2 in the cortex (“co”) and the epidermis (“ep”) in Col-0 (WT) and cmi1. Arrowheads highlight the basal localization of PIN2 in WT cortex and apical and apolar localization in cmi1 cortex. (B) Quantitative analysis of PIN2 distribution. Scale bar 20 μm. Error bars SE. Underlying data for this figure can be found in S2 Data. cmi1, Ca2+-dependent modulator of ICR1; Col-0, Columbia-0; PIN, PINFORMED; WT, wild type. (TIF) [file pbio.3000085.s005.tif]

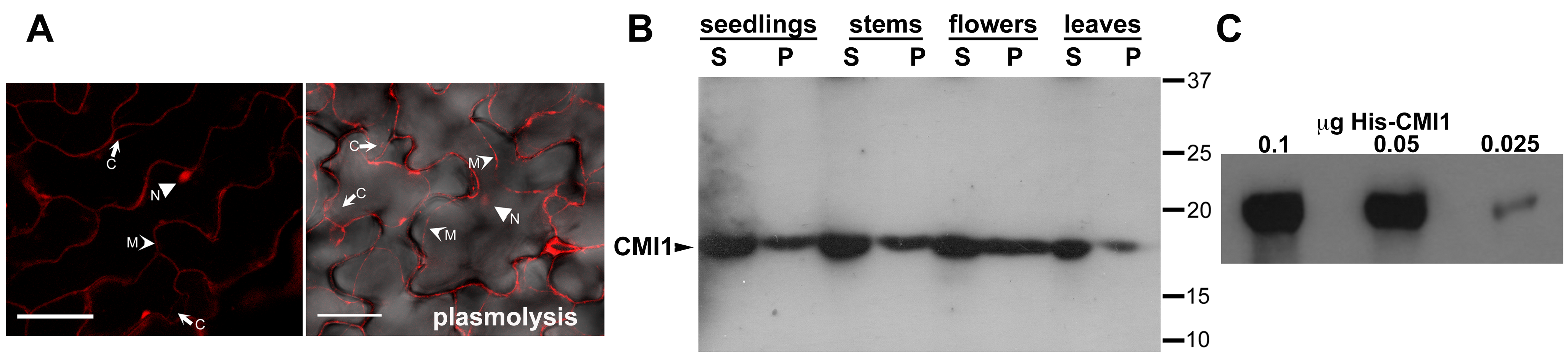

Supplement: S6 Fig — (A) Subcellular localization of mRFP-CMI1 in Arabidopsis cotyledon pavement cells. Localization of mRFP-CMI1 in the plasma membrane can be seen following plasmolysis (right panel). (B) Protein immunoblot decorated with anti-CMI1 antibodies showing the distribution of CMI1 between the soluble and insoluble fraction in the specified tissue samples. (C) The sensitivity of the anti-CMI1 antibodies as determined by protein immunoblot of the specified amounts of His6-CMI1. CMI1, Ca2+-dependent modulator of ICR1; mRFP, monomeric red fluorescent protein. (TIF) [file pbio.3000085.s006.tif]

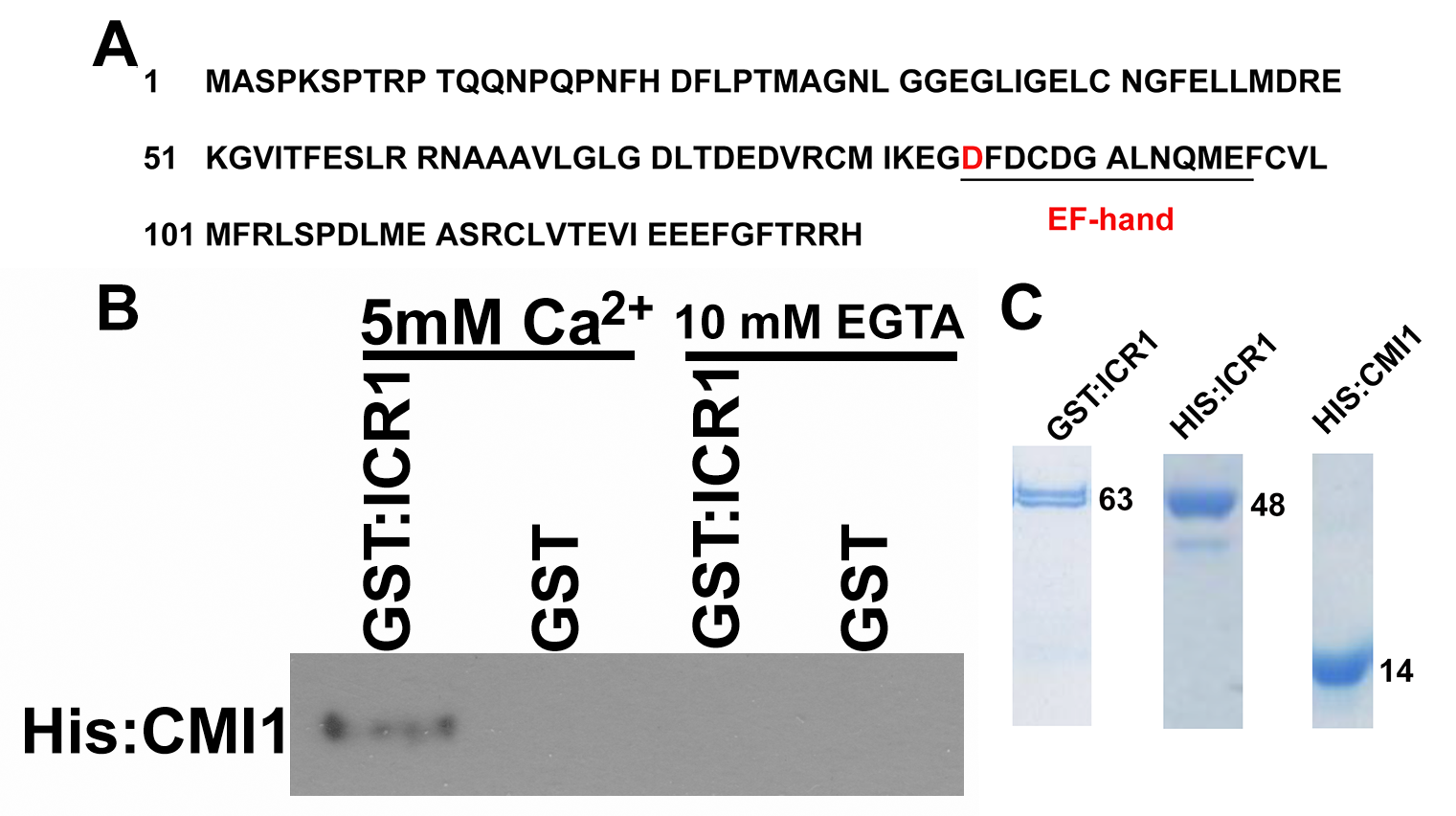

Supplement: S7 Fig — (A) The amino acid sequence of CMI1. The loop region of the single EF-hand is underlined, and the D85 critical for Ca2+ binding is highlighted in red. (B) Protein immunoblot decorated with anti-poly-His antibodies showing that pull-down of His6-CMI1 by GST-ICR1 is specific and Ca2+-dependent. (C) Coomassie brilliant blue–stained SDS-polyacrylamide gel showing specified E. coli expressed and purified recombinant proteins used for the pull-down and immunoprecipitation assays (Fig 1). Numbers denote Mr in kDa. CMI1, Ca2+-dependent modulator of ICR1; ICR1, interactor of constitutively active ROP. (TIF) [file pbio.3000085.s007.tif]

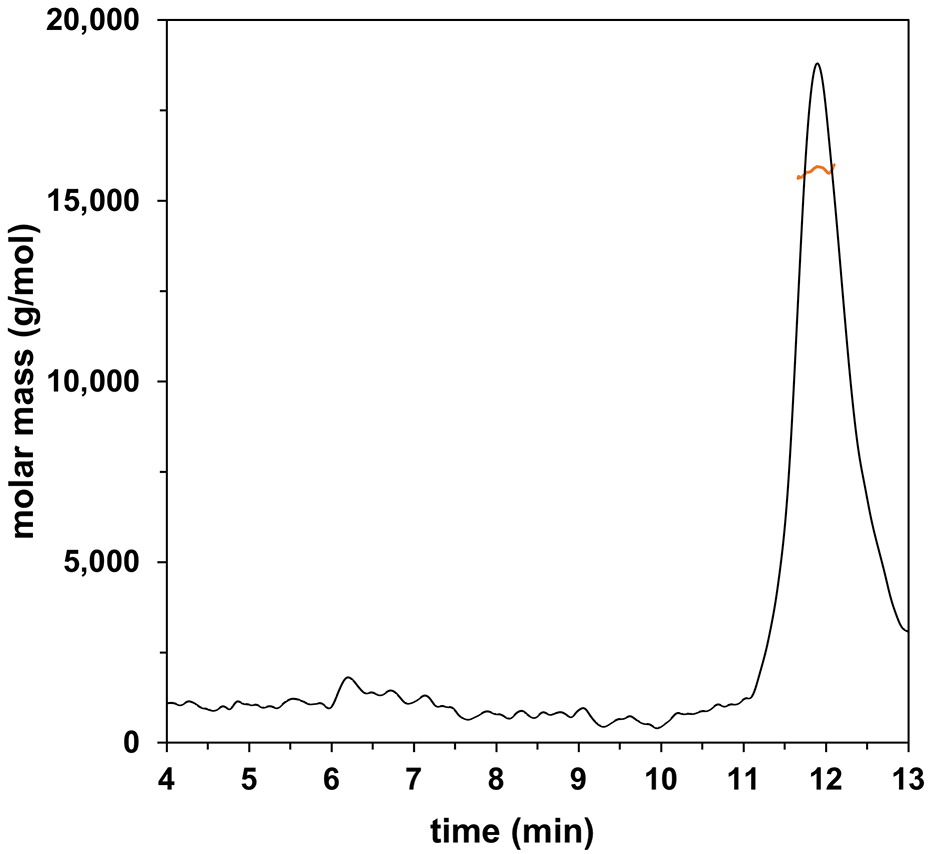

Supplement: S8 Fig — An SEC-MALS elution profile of 2 μg CMI1 in 2 mM Ca2+ solution. CMI1 eluted as a single peak with a molecular mass (red line) corresponding to a monomeric form. The profile is identical to that obtained with 4 μg protein (Fig 2E). Underlying data for this figure can be found in S2 Data. CMI1, Ca2+-dependent modulator of ICR1; SEC-MALS, size-exclusion chromatography multiangle light scattering. (TIF) [file pbio.3000085.s008.tif]

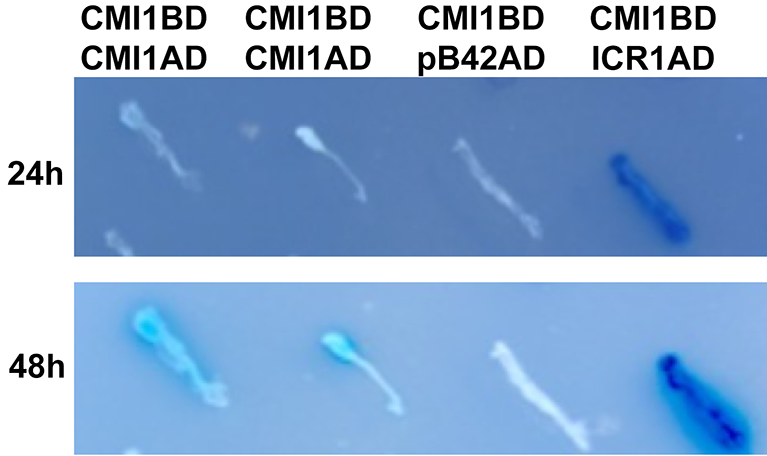

Supplement: S9 Fig — Yeast two-hybrid assays were carried out in the LexA system. In CMI1 self-interaction assays, weak X-Gal activity was evident after 48 hours. Strong X-Gal activity was observed in assays with ICR1 and no activity with the vector control. CMI1, Ca2+-dependent modulator of ICR1; ICR1, interactor of constitutively active ROP. (TIF) [file pbio.3000085.s009.tif]

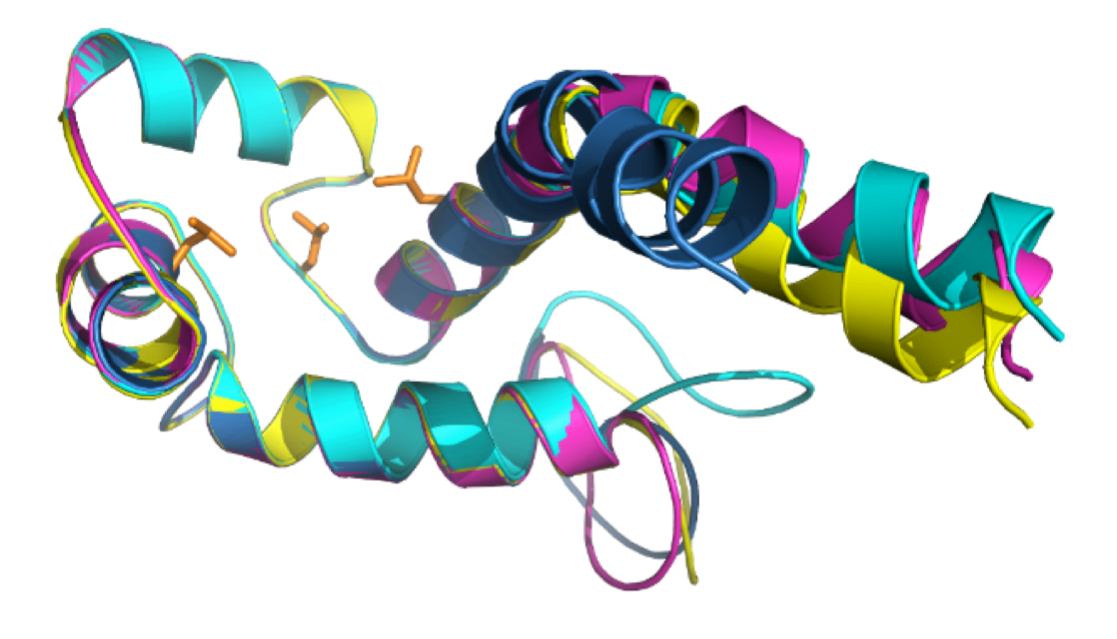

Supplement: S10 Fig — Structural models of CMI1 (blue) aligned with structural models of its hydrophobic pocket mutants; L59A (cyan), L92A (magenta), and L100A (yellow). The structure of main core of the CMI1 containing its hydrophobic pocket is not affected by the mutated residues (orange sticks). The structural deviations in the N- and C-terminal domains likely result from the low confidence of the model in these regions of the protein. CMI1, Ca2+-dependent modulator of ICR1. (TIF) [file pbio.3000085.s010.tif]

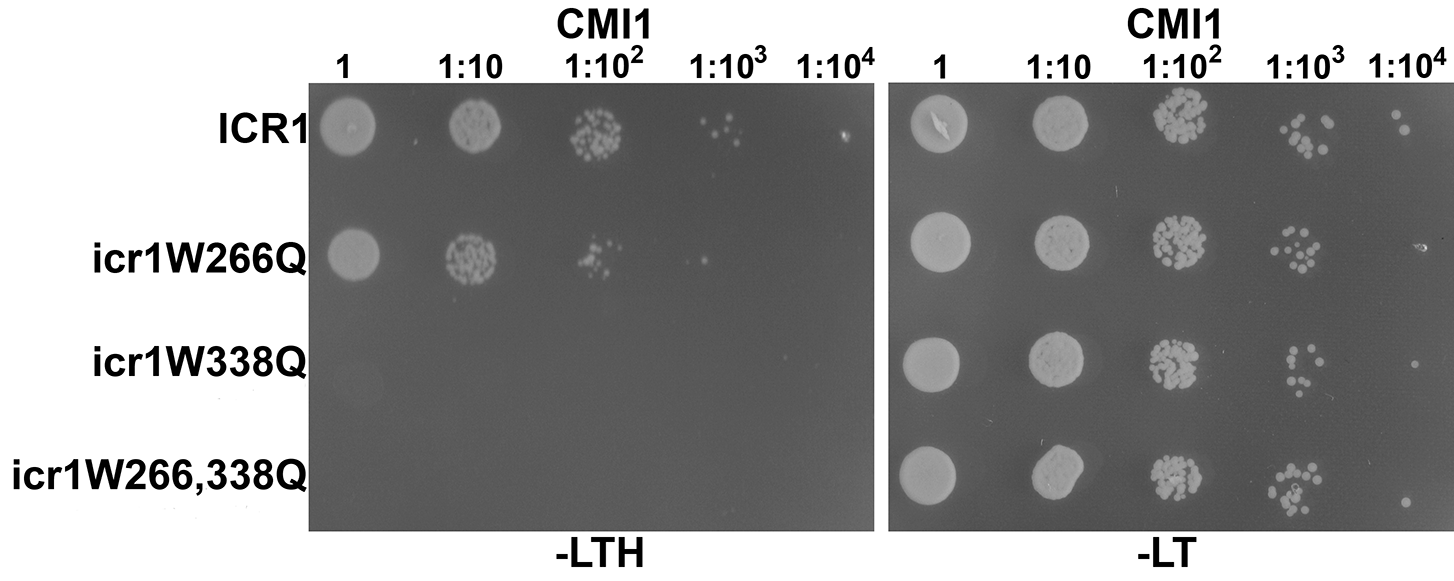

Supplement: S11 Fig — Numbers above the panel denote dilution order. -LT, Leu- and Trp-dropout medium; -LTH: Leu-, Trp-, and His-dropout medium. (TIF) [file pbio.3000085.s011.tif]

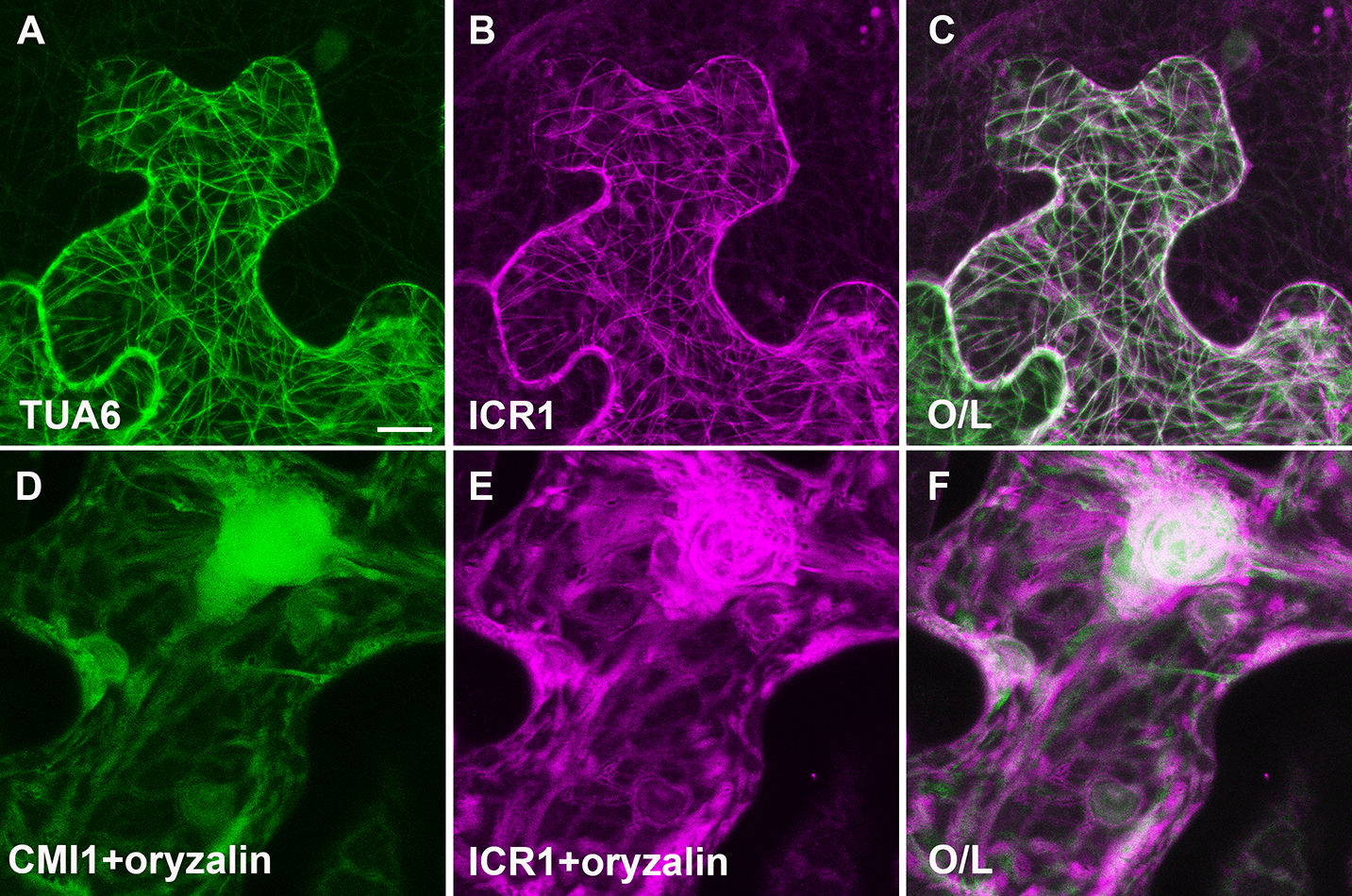

Supplement: S12 Fig — (A-C) ICR1-mCherry (ICR1) is colocalized to MTs with TUA6-GFP MT marker on MTs. (D-F) Localization of ICR1 and GFP-CMI1 (CMI1) on MTs is sensitive to the anti-MT drug oryzalin. O/L mCherry/GFP overlay. Bar: 20 μm. CMI1, Ca2+-dependent modulator of ICR1; GFP, green fluorescent protein; ICR1, interactor of constitutively active ROP; MT, microtubule; TUA6, Tubulin alpha-6. (TIF) [file pbio.3000085.s012.tif]
