# Supplementary material for: A novel Ca2+-binding protein that can rapidly transduce auxin responses during root growth
Source: PLoS Biol. 2019 Jul 11;17(7):e3000085. doi: 10.1371/journal.pbio.3000085 (PMC6650080; doi:10.1371/journal.pbio.3000085)
Supplement: S1 Table — (DOCX) [file pbio.3000085.s013.docx]

**S1 Table. Plasmids used in this study**

|  | ***Name*** | ***Description*** | ***E. coli*** | ***S. cerevisiae*** | ***Plant*** | **Source** |
| --- | --- | --- | --- | --- | --- | --- |
| 1 | *pSY1729* | *pGBT-ICR1* |  | + |  | [2] |
| 2 | *pSY1760* | *pGBT-ICR2* |  | + |  | [2] |
| 3 | *pSY611* | *pGBT-ICR4* |  | + |  | This work |
| 4 | *pSY1565* | *pGAD-CMI1* |  | + |  | This work |
| 5 | *pSY1553* | *pGAD-cmi1D85N* |  | + |  | This work |
| 6 | *pSY1317* | *pET-28a-ICR1-GST* | + |  |  | [2] |
| 7 | *pSY649* | *pET-28a-CMI1-His* | + |  |  | This work |
| 8 | *pSY468* | *pET-28a-ICR1-His* | + |  |  | This work |
| 9 | *pSY1351* | *pCAMBIA_2300_-35S::mRFP-CMI1* |  |  | + | This work |
| 10 | *pSY1804* | *pMDC162-pCMI1::CMI1-GUS* |  |  | + | This work |
| 11 | *pSY1806* | *pART27-pCMI1::LhG4* |  |  | + | This work |
| 12 | *pSY1807* | *pMLBART-10Xop::mRFP-CMI1* |  |  | + | This work |
| 13 | *pSY1569* | *pUBQ10::YC3.6 (Yellow Cameleon)* |  |  | + | [1] |
| 14 | *pSY2400* | *pEntry221-CMI1* | + |  |  | This work |
| 15 | *pSY2402* | *pExpression22-CMI1* | + | + |  | This work |
| 16 | *pSY2404* | *pExpression32-CMI1* | + | + |  | This work |
| 17 | *pSY2406* | *pJET1.2-BamHI-CMI1-NotI* | + |  |  | This work |
| 18 | *pSY2408* | *pET21d-Hisx8-TEV-CMI1* | + |  |  | This work |
| 19 | *pSY2424* | *pEntry221-ICR1* | + |  |  | This work |
| 20 | *pSY2425* | *pEntry221-icr1 51-344aa* | + |  |  | This work |
| 21 | *pSY2426* | *pEntry221-icr1 101-344aa* | + |  |  | This work |
| 22 | *pSY2427* | *pEntry221-icr1 151-344aa* | + |  |  | This work |
| 23 | *pSY2428* | *pEntry221-icr1 201-344aa* | + |  |  | This work |
| 24 | *pSY2429* | *pEntry221-icr1 251-344aa* | + |  |  | This work |
| 25 | *pSY2430* | *pEntry221-icr1 301-344aa* | + |  |  | This work |
| 26 | *pSY2431* | *pEntry221-icr1 1-300aa* | + |  |  | This work |
| 27 | *pSY2432* | *pEntry221-icr1 1-250aa* | + |  |  | This work |
| 28 | *pSY2433* | *pEntry221-icr1 1-200aa* | + |  |  | This work |
| 29 | *pSY2434* | *pEntry221-icr1 1-150aa* | + |  |  | This work |
| 30 | *pSY2435* | *pEntry221-icr1 1-100aa* | + |  |  | This work |
| 31 | *pSY2436* | *pEntry221-icr1 1-50aa* | + |  |  | This work |
| 32 | *pSY2437* | *pExpression32-ICR1* | + | + |  | This work |
| 33 | *pSY2438* | *pExpression32-icr1 51-344aa* | + | + |  | This work |
| 34 | *pSY2439* | *pExpression32-icr1 101-344aa* | + | + |  | This work |
| 35 | *pSY2440* | *pExpression32-icr1 151-344aa* | + | + |  | This work |
| 36 | *pSY2441* | *pExpression32-icr1 201-344aa* | + | + |  | This work |
| 37 | *pSY2442* | *pExpression32-icr1 251-344aa* | + | + |  | This work |
| 38 | *pSY2443* | *pExpression32-icr1 301-344aa* | + | + |  | This work |
| 39 | *pSY2444* | *pExpression32-icr1 1-300aa* | + | + |  | This work |
| 40 | *pSY2445* | *pExpression32-icr1 1-250aa* | + | + |  | This work |
| 41 | *pSY2446* | *pExpression32-icr1 1-200aa* | + | + |  | This work |
| 42 | *pSY2447* | *pExpression32-icr1 1-150aa* | + | + |  | This work |
| 43 | *pSY2448* | *pExpression32-icr1 1-100aa* | + | + |  | This work |
| 44 | *pSY2449* | *pExpression32-icr1 1-50aa* | + | + |  | This work |
| 45 | *pSY2450* | *pEntry221-icr1 W266Q* | + |  |  | This work |
| 46 | *pSY2451* | *pEntry221-icr1 W338Q* | + |  |  | This work |
| 47 | *pSY2452* | *pEntry221-icr1 W266,338Q* | + |  |  | This work |
| 48 | *pSY2453* | *pEntry221-icr1 W266A* | + |  |  | This work |
| 49 | *pSY2454* | *pEntry221-icr1 W338A* | + |  |  | This work |
| 50 | *pSY2455* | *pEntry221-icr1 W266,338A* | + |  |  | This work |
| 51 | *pSY2456* | *pExpression32-icr1 W266Q* | + | + |  | This work |
| 52 | *pSY2457* | *pExpression32-icr1 W338Q* | + | + |  | This work |
| 53 | *pSY2458* | *pExpression32-icr1 W266,338Q* | + | + |  | This work |
| 54 | *pSY2459* | *pExpression32-icr1 W266A* | + | + |  | This work |
| 55 | *pSY2460* | *pExpression32-icr1 W338A* | + | + |  | This work |
| 56 | *pSY2461* | *pExpression32-icr1 W266,338A* | + | + |  | This work |
| 57 | *pSY2490* | *pEntry221-cmi1 D85N* | + |  |  | This work |
| 58 | *pSY2492* | *pExpression22-cmi1 D85N* | + | + |  | This work |
| 59 | *pSY2494* | *pExpression32-cmi1 D85N* | + | + |  | This work |
| 60 | *pSY2812* | *pEntry221-cmi1 L59A* | + |  |  | This work |
| 61 | *pSY2813* | *pEntry221-cmi1 L92A* | + |  |  | This work |
| 62 | *pSY2814* | *pEntry221-cmi1 L100A* | + |  |  | This work |
| 63 | *pSY2818* | *pExpression22-cmi1 L59A* | + | + |  | This work |
| 64 | *pSY2819* | *pExpression22-cmi1 L92A* | + | + |  | This work |
| 65 | *pSY2820* | *pExpression22-cmi1 L100A* | + | + |  | This work |
| 66 | *pSY2872* | *pGWB6-35S::eGFP-CMI1* | + |  | + | This work |
| 67 | *pSY2873* | *pGWB6-35S::eGFP-cmi1 D85N* | + |  | + | This work |
| 68 | *pSY2874* | *pGWB6-35S::eGFP-cmi1 L59A* | + |  | + | This work |
| 69 | *pSY2888* | *pEntry221-icr1 W338A without stop codon* | + |  |  | This work |
| 70 | *pSY2892* | *pB7m34GW-35S::icr1 W338A-mCherry* | + |  | + | This work |
| 71 | *pSY416* | *pEntry221-ICR1 without stop codon* | + |  |  | Hasana |
| 72 | *pSY480* | *pB7m34GW-35S::ICR1-mCherry* | + |  | + | Hasana |
| 73 | *pSY1776* | *pCAMBIA3300-GFP-TUA6* | + |  | + | Mirav |
