# Supplementary material for: A novel Ca2+-binding protein that can rapidly transduce auxin responses during root growth
Source: PLoS Biol. 2019 Jul 11;17(7):e3000085. doi: 10.1371/journal.pbio.3000085 (PMC6650080; doi:10.1371/journal.pbio.3000085)
Supplement: S2 Table — (DOCX) [file pbio.3000085.s014.docx]

**S2 Table. Materials used in this work**

|  | **Product name** | **Application** | **Company name** | **Catalog number** |
| --- | --- | --- | --- | --- |
| **A** | Phusion High-Fidelity DNA Polymerase | High-fidelity PCR cloning | Thermo Scientific | F-530S |
| **B** | *PfuUltra* High-Fidelity DNA Polymerase | Site directed mutagenesis | Agilent Technologies | 600380 |
| **C** | *PfuUltra* II Fusion HS DNA Polymerase | Site directed mutagenesis | Agilent Technologies | 600670 |
| **D** | Taq Ready Mix | Colony PCR (bacteria and yeast) | Hy-labs | EZ-3007 |
| **E** | *DpnI* restriction enzyme | Eliminate unwanted templates after SDM and TPCR | Thermo Scientific | ER1701 |
| **F** | *BamHI* restriction enzyme | Restriction enzyme cloning | Thermo Scientific | ER0051 |
| **G** | *NdeI* restriction enzyme | Restriction enzyme cloning | Thermo Scientific | ER0581 |
| **H** | *NotI* restriction enzyme | Restriction enzyme cloning | Thermo Scientific | ER0591 |
| **I** | FastDigest *NotI* restriction enzyme | Restriction enzyme cloning | Thermo Scientific | FD0593 |
| **J** | SacI restriction enzyme | Restriction enzyme cloning | Thermo Scientific | ER1131 |
| **K** | XhoI restriction enzyme | Restriction enzyme cloning | Thermo Scientific | ER0691 |
| **L** | FastAP Thermosensitive Alkaline Phosphatase | Dephosphorylation of cloning vector to prevent recircularization during ligation | Thermo Scientific | EF0654 |
| **M** | T4 DNA Ligase | Ligation of DNA fragments generated by restriction enzymes | Thermo Scientific | EL0011 |
| **N** | T4 DNA Ligase | Ligation of DNA fragments generated by restriction enzymes (for difficult reactions) | NEB | M0202T |
| **O** | CloneJET PCR Cloning Kit | Cloning of PCR products | Thermo Scientific | K1231 |
| **P** | Gateway BP Clonase II Enzyme Mix | BP recombination reaction | Invitrogen | 11789-020 |
| **Q** | Gateway LR Clonase II Enzyme Mix | LR recombination reaction | Invitrogen | 11791-020 |
| **R** | Gateway LR Clonase II Plus Enzyme Mix | MultiSite LR recombination reaction | Invitrogen | 12538-120 |
| **S** | QIAEX II Gel Extraction Kit | Gel extraction of DNA fragments | QIAGEN | 20021 |
| **T** | Wizard SV Gel and PCR Clean-Up System | Gel extraction of DNA fragments and purification of PCR products | Promega | A9281 |
| **U** | DNA-spin Plasmid DNA Purification Kit | Purification of bacteria plamid DNA | iNtRON Biotechnology | 17096 |
| **V** | AccuPrep Plasmid Mini Extraction Kit | Purification of bacteria plamid DNA | BIONEER | K-3030 |
| **W** | GenElute Plant Genomic DNA Miniprep Kit | Elicitation of plants DNA | Sigma-Aldrich | G2N70-1KT |
| **X** | RNeasy Plus Mini Kit | Elicitation of plants total RNA | QIAGEN | 74134 |
| **Y** | High Capacity cDNA Reverse Transcription Kit with RNase Inhibitor | Reverse transcription of mRNA to single-stranded cDNA | Applied Biosystems | 4374966 |
| **Z** | Yeast Synthetic Drop-out Media Supplements without leucine and tryptophan | Preparation of -LT yeast growth media | Sigma-Aldrich | Y0750 |
| **A*** | Yeast Synthetic Drop-out Media Supplements without histidine, leucine and tryptophan | Preparation of -LTH yeast growth media | Sigma-Aldrich | Y2146 |
| **B*** | Yeast Synthetic Drop-out Media Supplements without leucine | Preparation of -L yeast growth media | Sigma-Aldrich | Y1376 |
| **C*** | Yeast Synthetic Drop-out Media Supplements without tryptophan | Preparation of -T yeast growth media | Sigma-Aldrich | Y1876 |
