# Supplementary material for: A novel Ca2+-binding protein that can rapidly transduce auxin responses during root growth
Source: PLoS Biol. 2019 Jul 11;17(7):e3000085. doi: 10.1371/journal.pbio.3000085 (PMC6650080; doi:10.1371/journal.pbio.3000085)
Supplement: S3 Table — (DOCX) [file pbio.3000085.s015.docx]

**S3 Table. *Arabidopsis thaliana* lines used in this study**

|  | **Name** | **Source** |
| --- | --- | --- |
| 1 | *WT (Col-0)* | SALK Institute |
| 2 | *WT (Ler)* | SALK Institute |
| 3 | *axr1* | [1] |
| 4 | *cmi1(Ler)* | GT_24505 CSHL collection |
| 5 | *DR5::GFP_rev_* in *Col-0* | [2] |
| 6 | *DR5::GFP_rev_* in *Ler (crosses)* | This work |
| 7 | *DR5::GFP_rev_* in *cmi1 (crosses)* | This work |
| 8 | *pICR1>>GFP-ICR1* in *Col-0* | [3] |
| 9 | *pICR1>>GFP-ICR1* in *Ler (crosses)* | This work |
| 10 | *pICR1>>GFP-ICR1* in *cmi1 (crosses)* | This work |
| 11 | *pUBQ10::YC3.6* in *Ler (transformation)* | This work |
| 12 | *pUBQ10::YC3.6* in *cmi1 (transformation)* | This work |
| 13 | *pCMI1>>mRFP-CMI1 (crosses)* | This work |
| 14 | *pICR1>>mRFP-CMI1 (crosses)* | This work |
| 16 | *pCMI2>>GFP-ICR1* | [4] |
| 18 | *pCMI1::CMI1-GUS* in *Col-0* | This work |
| 19 | *pCMI1::CMI1-GUS* in *cmi1 (crosses)* | This work |

**References**

1. Lincoln C, Britton JH, Estelle M. Growth and development of the axr1 mutants of Arabidopsis. Plant Cell. 1990;2(11):1071-80. doi: 10.1105/tpc.2.11.1071. PubMed PMID: 1983791; PubMed Central PMCID: PMC159955.

2. Friml J, Vieten A, Sauer M, Weijers D, Schwarz H, Hamann T, et al. Efflux-dependent auxin gradients establish the apical-basal axis of Arabidopsis. Nature. 2003;426(6963):147-53. Epub 2003/11/14. doi: 10.1038/nature02085. PubMed PMID: 14614497.

3. Hazak O, Bloch D, Poraty L, Sternberg H, Zhang J, Friml J, et al. A rho scaffold integrates the secretory system with feedback mechanisms in regulation of auxin distribution. PLoS Biol. 2010;8(1):e1000282. Epub 2010/01/26. doi: 10.1371/journal.pbio.1000282. PubMed PMID: 20098722; PubMed Central PMCID: PMC2808208.

4. Hazak O, Obolski U, Prat T, Friml J, Hadany L, Yalovsky S. Bimodal regulation of ICR1 levels generates self-organizing auxin distribution. Proc Natl Acad Sci U S A. 2014;111(50):E5471-9. doi: 10.1073/pnas.1413918111. PubMed PMID: 25468974; PubMed Central PMCID: PMC4273421.
